# Women carry the weight of deprivation on physical inactivity: Moderated mediation analyses in a European sample of adults over 50 Years of age

**DOI:** 10.1016/j.ssmph.2022.101272

**Published:** 2022-10-23

**Authors:** Silvio Maltagliati, Ilyes Saoudi, Philippe Sarrazin, Stéphane Cullati, Stefan Sieber, Aïna Chalabaev, Boris Cheval

**Affiliations:** a Univ. Grenoble Alpes, SENS, F-38000, Grenoble, France; b Population Health Laboratory, University of Fribourg, Switzerland; c Department of Readaptation and Geriatrics, University of Geneva, Switzerland; d LIVES Centre, Swiss Centre of Expertise in Life Course Research, University of Lausanne, Switzerland; e Swiss Center for Affective Sciences, University of Geneva, Geneva, Switzerland; f Laboratory for the Study of Emotion Elicitation and Expression (E3Lab), Department of Psychology, University of Geneva, Geneva, Switzerland

**Keywords:** Physical activity, Deprivation, Body mass index, Gender, Intersectional approach

## Abstract

Deprived people are less physically active than privileged individuals. However, pathways underlying the association between deprivation and physical activity remain overlooked. We examined whether the association between deprivation and physical activity was mediated by body mass index (BMI). Consistent with an intersectional perspective (how the combination of belongingness to vulnerable social categories widens inequalities), we tested whether gender moderated this mediating pathway and hypothesized that the mediating effect of BMI would be stronger among women (vs men). Large-scale longitudinal data from 20,961 adults 50 years of age or older (57% women) from the Survey of Health, Ageing and Retirement in Europe were used. Social and material deprivation were measured by questionnaire, BMI and physical activity were reported from two to six years later. Simple mediation models showed that BMI partly mediated the association of material (total effect *c* = -0.14, proportion of mediated effect = 8%) and of social deprivation (*c* = -0.24, proportion of mediated effect = 4%) with physical activity. Moderated mediation models revealed that this mediating pathway was moderated by gender. The effect of deprivation on BMI was stronger among women (vs men), with BMI mediating 18% and 7% of the association of material and social deprivation with physical activity among women (vs 4% and 2% among men). Lower levels of physical activity observed among deprived older adults could be partly attributed to a higher BMI. Critically, this mechanism was exacerbated among women, reinforcing the need to understand how deprivation and gender interact to predict health behaviors.

## Highlights

• Body mass index mediates the association of material and social deprivation with physical activity.
• This mediating pattern is more pronounced among women, relative to men.
• The association between deprivation and a higher body mass index is exacerbated among women, compared to men.

In most developed countries, a significant decrease in mortality has been observed across the twentieth century (Oeppen & Vaupel, 2002). Yet, this trend is not equally distributed across the population (Lallo & Raitano, 2018; Remund et al., 2019) – the health gap between advantaged and disadvantaged individuals has at best maintained over time, or rather widened for some outcomes (Dowd et al., 2011; Tawiah et al., 2022). Disparities in the adoption of health behaviors at older age, such as physical activity, may partially explain why social health inequalities remain broad (Petrovic et al., 2018; Stringhini, 2010). Indeed, the benefits of being physically active in old age are numerous, such as improved longevity, musculoskeletal, muscular and immune health, improved emotional and cognitive health, as well as an overall reduced risk of all-cause mortality (Basso & Suzuki, 2017; Duggal et al., 2019; Felez-Nobrega et al., 2021; Paluch et al., 2022; Rhodes et al., 2017). Yet, compared to advantaged populations, disadvantaged ones consistently report lower levels of physical activity during leisure time (O’Donoghue et al., 2020), which ultimately reinforces socioeconomic health inequalities at older age (Khalatbari-Soltani et al., 2020; Ramsay et al., 2008; Shaw et al., 2014). In the context of an ageing population in developed countries (Christensen et al., 2009) and in light of the costs of physical inactivity on health care systems (Ding et al., 2016), understanding how socio-economic circumstances relate to physical activity levels among older adults is warranted.

Amongst the socio-economic circumstances linked with physical inactivity is deprivation, a multidimension construct which is defined as a deficiency in basic material and social needs (Myck et al., 2015). It encapsulates, but does not restrict to, traditional indicators of socio-economic circumstances (e.g., income, occupation, education), by focusing on access to material (i.e., access to goods) and social opportunities (i.e., access to social support), over and beyond individuals’ socio-economic position. Previous research established that older deprived people were less physically active than more privileged individuals during leisure time (Annear et al., 2009; Fox et al., 2011; Lang et al., 2008) (see also Farrell et al., 2014 among middle-aged adults). Yet, the pathways through which deprivation relates to lower physical activity remain overlooked (Diez Roux, 2016). Beyond environmental (e.g., access to facilities, walkability or adverse neighborhood conditions) (Hillsdon et al., 2007; Li et al., 2005), cultural (e.g., continuity of class habitus in older age) (Bourdieu, 1979; Cockerham et al., 2020; Pampel et al., 2010), or cognitive factors (e.g., motivations, cognitive functions) (Cheval et al., 2019; Hagger & Hamilton, 2020), body mass index (BMI) stands as one potential mediating candidate.

On the one hand, deprivation has been found to be associated with a higher BMI (Bann et al., 2017; Pruchno et al., 2014). Indeed, deprived obesogenic environments encourage higher food intake and restrict the access to affordable and nutritious food (Atanasova et al., 2022). Moreover, while cognitive functions play a critical role in health-behaviors trajectories at older age (Cheval et al., 2020), perceiving deprivation can tax the cognitive ‘bandwidth’, which leads to a weaker ability to self-regulate one's behavior and to resist giving into unhealthy food consumption (Beenackers et al., 2018; Mesler et al., 2022). On the other hand, though bi-directional associations between physical activity and BMI are proposed, a higher BMI has been repeatedly associated with a lower participation in physical activity (e.g., Cassidy et al., 2017; Ekelund et al., 2017). Nonetheless, whether BMI mediates the association between deprivation and physical activity has never been formally tested – a first knowledge gap that this study aims to fill.

Beyond the effect of deprivation on physical activity through BMI, an intersectional perspective further suggests that socio-economic factors may combine and that such combinations may reinforce inequalities in health behaviors (Bauer, 2014; Bowleg, 2012). For example, socioeconomic status and gender were found to interact when predicting health condition and physical activity, with the detrimental effects of a disadvantaged socio-economic background on health condition (Mackenbach et al., 1999) and on physical activity (Chalabaev et al., 2022; Cheval et al., 2018) being especially pronounced among older women. Here, we postulate that gender could moderate the mediating pathway between deprivation, BMI and physical activity in two distinct ways. First, gender may moderate the association between deprivation and BMI (i.e., first component of the mediating pathway) as, relative to men, the association between deprivation and BMI has been shown to be exacerbated among middle-aged and older women (Bann et al., 2017; Matheson et al., 2008; Shaw et al., 2014). Second, gender may also moderate the relationship between BMI and physical activity (i.e., second component of the mediating pathway): a higher BMI may represent a stronger barrier to physical activity among women, relative to men. Particularly, older women with a higher BMI are more exposed to stigma than their male counterparts (Andreyeva et al., 2008), which may in turn restrain their engagement in physical activity (Jackson & Steptoe, 2017) (see also Rojas-Sánchez et al., 2021; Vartanian & Shaprow, 2008 among middle-aged adults). However, at the time of writing, no study has investigated whether and how gender moderates the mediation between deprivation, BMI and physical activity among older adults.

The aim of the current study was twofold. First, we aimed to investigate whether BMI mediates the association between deprivation and physical activity. Second, we aimed to test whether gender moderates this mediating pathway. We hypothesized that BMI would mediate the association between deprivation and physical activity (H1). We further hypothesized that gender would moderate this mediating pathway – with the mediating role of BMI on the relationship between deprivation and physical activity being stronger among women than among men (H2). Accordingly, we examined whether gender moderated the association between deprivation and BMI (i.e., first component of the mediating pathway) and/or the association of BMI with physical activity (i.e., second component of the mediating pathway). To test these hypotheses, we relied on a large-scale sample composed of older European adults, which are at particular risk of physical inactivity.

## 1 Methods

### 1.1 Participants and procedure

Data were drawn from the Survey of Health, Ageing and Retirement in Europe (SHARE), a longitudinal population-based study on ∼140,000 European adults 50 years of age or older. Data related to health, environmental and socio-economic circumstances were measured every two years in eight waves between 2004 and 2019. Answers of participants living in 28 European countries and Israel were repeatedly collected using computer-assisted personal interviewing (CAPI) in their home. The relevant ethics committees in the participating countries approved SHARE and all participants provided written informed consent. All procedures and measures of SHARE were described in detail elsewhere (Börsch-Supan et al., 2013).

To be included in the study, participants had to be between 50 years and 96 years old and had completed measures of deprivation in Wave 5 (2013), of BMI in Wave 6 (2015) and of physical activity in Wave 7 (2017) or in Wave 8 (2019), as well as of confounding variables (see Fig. S1 for a flowchart). In Wave 5 , material and social deprivation were assessed using a devoted set of items. Two years later, in Wave 6, BMI was measured. In Waves 7 and 8 (i.e., two or four years after assessing BMI, respectively) physical activity was measured. However, in Wave 7, participants who did not complete the so-called SHARELIFE questionnaire (a specific type of questionnaire focused on life history events) in Wave 3 answered the SHARELIFE questionnaire. For those participants, the main questionnaire including the measure of physical activity status was skipped. To minimize the resulting loss of participants and to increase the sample size, we also included participants who reported physical activity in Wave 8. For participants having a physical activity measure both in Waves 7 and 8, we included the most recent measure of physical activity (i.e. in Wave 8). Otherwise, for those who had this information available only in either Wave 7 or Wave 8, we included the information from the respective wave. In the final sample, physical activity was measured in Wave 8 for 17,944 participants and in Wave 7 for 3017 participants, of the 20,961 included in the study (see robustness analyses).

### 1.2 Measures

***Predictors:****Material and social deprivation* were measured by a questionnaire in Wave 5, with items being specific to the target age group of the SHARE survey (Bertoni et al., 2015). For material deprivation, participants answered on 11 items focusing on different dimensions of the economic circumstances of households (e.g., affording to buy fruit and meat, necessary groceries, clothes, shoes, to go on holidays, to receive health care). An aggregated weighted indicator of material deprivation was computed by averaging the different items (Bertoni et al., 2015). The score ranged from continuous values between 0 and 1, with higher values reflecting a higher material deprivation.

*For social deprivation*, participants answered on 15 items focusing on different dimensions of everyday life participation (e.g., skills in reading or in technologies), as well as on the perceived quality of the neighborhood (e.g., access to shops, banks and health structures, vandalism, helpfulness of people in the local area). Similar to material deprivation, items were averaged into a single weighted index of social deprivation (Myck et al., 2015). The score ranged from continuous values between 0 and 1, with higher values reflecting a higher social deprivation.

These indicators of perceived deprivation have been used to predict health-related outcomes, including perceived health status, difficulties in mobility, risks of mortality and well-being (Myck et al., 2020; Terraneo, 2021).

***Mediating variable****: BMI* was computed based on participants’ self-reported height and weight in Wave 6. It was expressed as a continuous variable, in kg/m^2^.

***Outcome****: Physical activity* was self-reported by questionnaire in Wave 7 and/or in Wave 8. As mentioned above, we included the most recent available measure of physical activity in the analyses. It was measured using the two following questions: “How often do you engage in vigorous physical activity, such as sports, heavy housework, or a job that involves physical labor?” and “How often do you engage in activities that require a low or moderate level of energy such as gardening, cleaning the car, or doing a walk?“. Participants answered using a four-point scale with the following options: *Hardly ever, or never*; *One to three times a month*; *Once a week*; *More than once a week*. Participants who did not answer “*More than once a week*” to either item were classified as physically inactive, while other participants were classified as physically active. As described in previous research (Cheval et al., 2018, 2019), this strategy reduces the potential misclassification bias which would lead to physically inactive participants being incorrectly classified as physically active.

This two-item scale has been found to be robustly associated with health outcomes, such as chronic diseases (de Souto Barreto et al., 2017), cognitive functions (Cheval et al., 2019, 2020), depressive symptoms (Boisgontier et al., 2020), diabetes (Cheval et al., 2021) or risks of hospitalization following COVID-19 infections (Maltagliati et al., 2021). Moreover, in the context of longitudinal large-scale surveys, similar one- or two-items scales have provided acceptable validity regarding the assessment of physical activity (Milton et al., 2011), including among older adults (Gill et al., 2012).

***Moderating variable****: Gender* was measured in Wave 5, with participants reporting being either “female” or “male”.

***Confounders*** included participants' age, country of residence, education, ability to make ends meet and occupation status. These confounders were selected after we had specified our conceptual model in which we aimed to identify variables that can potentially cause the predictor, the mediator and the outcome (Lipsky & Greenland, 2022). To decrease risk of bias in estimates, we did not include variables which were likely colliders (i.e., variables that potentially represent the consequence – rather than the cause – of the predictor, the mediator and the outcome, e.g., physical or mental health) (Lipsky & Greenland, 2022). Education (primary, secondary, tertiary, categories based on the International Standard Classification of Education) was measured when participants were first included in the study and corresponded to the highest attained level of education. Occupation status of participants' main job (never worked, low skill, high skill) was measured in the SHARELIFE questionnaire (i.e., in Wave 3 or in Wave 7 for participants who did not answer in Wave 3) (Sieber et al., 2022). As done in previous research (Cheval et al., 2018), the occupational position was coded on the basis of a reclassification of the 10 main occupational groups of the International Standard Classification of Occupations (ISCO) according to their skill levels. The first and second levels were classified as “low skill” occupational position, and the third and fourth levels were coded as “high skill” occupational position. Ability to make ends meet (with great difficulty, with some difficulty, fairly easily, easily) was measured in Wave 5 and referred to participants’ current perceived financial situation.

### 1.3 Statistical analyses

All analyses were computed on R ®, version 4.0.4. Continuous variables (i.e., material and social deprivation, BMI) were centered and scaled using the scale function of the R package psych (mean value of the sample = 0; standard-deviation = 1). Regarding categorical variables, physical activity (-0.5 for inactive individuals and 0.5 for active individuals) and gender (-0.5 for men and 0.5 for women) were contrast-coded.

Mediation models were computed through a two-step modeling strategy. In all models, material and social deprivation were separately included as predictors (with a medium-to-large correlation between material and social deprivation, *r* = 0.39, *p* < .001), alongside with confounding variables (age, country, education, ability to make ends, occupation status). A first set of linear models was fitted, with BMI (continuous variable, potential mediator) being specified as the dependent variable (Models 1a for material deprivation and 1b for social deprivation). Then, a second set of logistic models was fitted, with physical activity (dichotomous variable, outcome) being specified as the dependent variable (Models 2a for material deprivation and 2b for social deprivation). In the second set of models (Models 2a and 2b), BMI (potential mediator) was added as a predictor, alongside with material or social deprivation and confounding variables. To test the moderating effect of gender, the first and second sets of models were refitted in which we added gender and its interactive term with material and social deprivation on BMI (Models 3a and 3b) and with material, social deprivation and BMI on physical activity (Models 4a and 4b) as predictors. For linear models, we reported unstandardized beta coefficients (b), and for logistic models, we reported unstandardized beta coefficients (b), as well as odds-ratios (OR), which were calculated by exponentiating b.

To test the mediating pathway between deprivation, BMI and physical activity, we adopted two complementary approaches (Yzerbyt et al., 2018). First, as proposed by the index approach, mediation analyses were conducted based on the distribution-of-the product framework in order to estimate confidence intervals around the indirect effects (Hayes, 2015). Coefficients of material and social deprivation on BMI (*a* path) were multiplied by coefficients of BMI on physical activity (*b* path), with indirect effects and their 95% confidence intervals (i.e., indirect effect = *a* path × *b* path) being tested by bootstrapping this procedure (5000 simulations). A Monte Carlo 95% confidence interval (95% CI) around the indirect effect index which did not contain 0 is assumed to indicate a significant indirect effect. Second, as proposed by the component approach, to confirm the significance of the indirect effects, the two paths of indirect effects in Models 1 and Models 2 were examined. According to this approach, an indirect effect is supported when both paths are significant (*p* < .05) (Yzerbyt et al., 2018).

To examine the moderating effect of gender on this mediating pathway, we also relied on these two approaches. Specifically, as proposed by the index approach, we first tested whether the Monte Carlo 95% CI contained 0 for the index of the moderated indirect effect, with an interval not containing 0 being indicative of a significant moderated mediation. Then, as suggested by the component approach, we examined whether gender moderated the effect of deprivation on BMI (*a* path), the effect of BMI on physical activity (*b* path) or the effect of deprivation on physical activity (*c’* path) (Muller et al., 2005). Simple slopes analyses were further computed to decompose potential interactive effects.

Across these analyses, we also examined the direct effect of material and social deprivation on physical activity (*c’* paths), while controlling for BMI (potential mediator) and confounding variables. Total effects (*c* paths) were obtained from summing the indirect effects (i.e., *a* paths × *b* paths) and the direct effects (*c’* paths). Proportion of mediated effect was reported as an indicator for the effect size of the mediating pathway (Wen & Fan, 2015).

Mediation models were specified using the package mediation (Tingley et al., 2014) and were inspected using the package performance (i.e., linearity and normality of residuals, homogeneity of variance, undue influence).

### 1.4 Robustness analyses

In a first set of additional analyses, we added functional dependence in instrumental activities of daily living (IADL) and functional dependence in activities of daily living (ADL) at baseline (Wave 5) – two indicators of participants’ health status – as confounding variables.

In a second set of additional analyses, we tested whether the time of measurement of physical activity (i.e., Wave 7 versus Wave 8) was associated with BMI or with physical activity and influenced observed associations.

## 2 Results

In total, 20,961 individuals were included in the study (57% of women, mean age = 68 ± 9 years). In this sample, 14,760 individuals (70% of the full sample) were categorized as physically active. Moreover, 1980 participants (9% of the full sample) were classified as severely deprived (i.e., allocated above the 75th percentile of the full sample distribution in Wave 5 on both social and material deprivation). Regarding BMI, 7637 individuals (36% of the full sample) were classified as having a normal weight (i.e., BMI < 25 kg m^2^), 8696 as being overweight (41% of the full sample) (i.e., 25 kg m^2^ ≤ BMI < 30 kg m^2^) and 4628 as being obese (22% of the full sample) (i.e., BMI ≥ 30 kg m^2^). Descriptive statistics and univariate associations with physical activity are provided in Table 1, with material deprivation (*r* = -0.15, *p* < .001) and social deprivation (*r* = -0.21, *p* < .001) being both negatively correlated with being classified as physically active (vs inactive).

**Table 1:** Descriptive statistics and univariate associations.

N = 20,961	Mean (SD)	Range	*r*/χ^2^, *p*-value
Physical activity			
Physically active (N, %)	14,760 (70%)	–	–
Physically inactive (N, %)	6201 (30%)	–	–
**Material deprivation** (from 0 to 1)	0.12 (0.18)	0–1	*r* = -.15, *p* < .001
**Social deprivation** (from 0 to 1)	0.16 (0.13)	0–0.83	*r* = -.21, *p* < .001
**BMI**	27.00 (4.60)	13.62–74.74	*r* = -.10, *p* < .001
**Gender** (N, % of women)	11,898 (57%)	–	χ^2^ = 27.18, *p* < .001
**Age**	67.89 (8.76)	50–95	*r* = -.21, *p* < .001
**Country (N, %)**			
Austria	1197 (6%)	–	χ^2^ = 863.72, *p* < .001
Belgium	1914 (9%)	–	
Czech Republic	1787 (9%)	–	
Denmark	1720 (8%)	–	
Estonia	1802 (9%)	–	
France	1654 (8%)	–	
Germany	2308 (11%)	–	
Israël	347 (2%)	–	
Italy	1823 (9%)	–	
Luxembourg	424 (2%)	–	
Slovenia	952 (5%)	–	
Spain	1670 (8%)	–	
Sweden	1897 (9%)	–	
Switzerland	1466 (7%)	–	
**Education (N, %)**			
Primary school	3868 (19%)	–	χ^2^ = 570.78, *p* < .001
Secondary school	11814 (56%)	–	
Tertiary school	5279 (25%)	–	
**Ability to make ends meet (N, %)**			
Fairly easily	6344 (30%)	–	χ^2^ = 582.46, *p* < .001
Easily	9545 (46%)		
With some difficulty	3779 (18%)	–	
With great difficulty	1293 (6%)	–	
**Occupation status (N, %)**			
High skill	7348 (35%		χ^2^ = 343.16, *p* < .001
Low skill	12623 (60%)		
Never worked	990 (5%)		

*Note*. SD: standard deviation. Univariate associations with physical activity were computed using correlations (*r*) and chi-square tests (χ^2^) for continuous and categorical variables, respectively. For gender, being a man (vs a woman) was positively associated with odds of being classified as physically active (vs inactive).

### 2.1 Material deprivation

Results of the simple mediation model showed a significant indirect effect of material deprivation on physical activity through BMI (index of the indirect effect = -0.0023, 95% CI = [-0.0031; -0.0015], *p* < .001) (Table 2). Specifically, material deprivation was positively associated with BMI (Model 1a: b = 0.06, 95% CI = [0.04; 0.08], *p* < .001) and, in turn, higher BMI was associated with lower odds of being physically active (vs inactive) (Model 2a: b = -0.21, 95% CI = [-0.24;0.18], OR = 0.81, 95% CI = [0.78; 0.83], *p* < .001). The direct effect of material deprivation on physical activity remained significant after adjustment for BMI (Model 2a: b = -0.13, 95% CI = [-0.17; -0.09], OR = 0.88, 95% CI = [0.84; 0.92], *p* < .001). In sum, BMI partly mediated the association of material deprivation with physical activity (total effect *c* = -0.14, proportion of mediated effect = 8%).

**Table 2:** Estimates for all predictors in the models including material deprivation.

Predictors	Model 1a (BMI as outcome)			Model 3a (BMI as outcome)			Model 2a (PA as outcome)			Model 4a (PA as outcome)		
	b	95% CI	*p*	b	95% CI	*p*	OR	95% CI	*p*	OR	95% CI	*p*
Intercept	0.01	-0.06; 0.08	.775	0.00	-0.07; 0.07	.997	1.95	1.67; 2.27	<.001	1.94	1.67; 2.26	<.001
Material deprivation	0.06	0.04; 0.08	<.001	0.04	0.02; 0.06	<.001	0.88	0.84; 0.92	<.001	0.87	0.83; 0.91	<.001
BMI	–	–	–				0.81	0.78; 0.83	<.001	0.80	0.77; 0.83	<.001
Gender (ref: men)	–	–	–	-0.14	-0.17; -0.12	<.001	–	–	–	0.88	0.82; 0.94	<.001
Material deprivation × gender	–	–	–	0.14	0.11; 0.17	<.001	–	–	–	1.07	1.00; 1.14	.049
BMI × gender	–	–	–	–	–	–	–	–	–	1.04	0.97; 1.11	.230
Age	-0.07	-0.08; -0.05	<.001	-0.07	-0.09; -0.06	<.001	0.56	0.53; 0.58	<.001	0.55	0.53; 0.57	<.001
*Country (ref: Belgium)*												
Austria	0.11	0.04; 0.18	.002	0.13	0.06; 0.20	<.001	1.84	1.55; 2.18	<.001	1.86	1.57; 2.21	<.001
Czech Republic	0.30	0.23; 0.36	<.001	0.31	0.25; 0.37	<.001	1.26	1.09; 1.45	.002	1.27	1.10; 1.47	.001
Denmark	-0.06	-0.13; -0.00	.050	-0.06	-0.12; 0.01	.076	2.18	1.85; 2.57	<.001	2.19	1.86; 2.59	<.001
Estonia	0.19	0.12; 0.26	<.001	0.20	0.14; 0.27	<.001	1.43	1.23; 1.67	<.001	1.45	1.25; 1.69	<.001
France	-0.10	-0.16; -0.03	.003	-0.09	-0.16; -0.03	.005	1.13	0.97; 1.31	.108	1.13	0.98; 1.31	.095
Germany	0.12	0.06; 0.18	<.001	0.13	0.07; 0.19	<.001	1.48	1.28; 1.70	<.001	1.48	1.29; 1.70	<.001
Israël	0.13	0.02; 0.24	.026	0.14	0.03; 0.25	.015	0.82	0.64; 1.05	.114	0.83	0.65; 1.06	.128
Luxembourg	-0.20	-0.26; -0.13	<.001	-0.20	-0.27; -0.14	<.001	0.76	0.66; 0.88	<.001	0.76	0.66; 0.88	<.001
Slovenia	0.09	-0.01; 0.20	.071	0.09	-0.01; -0.19	.086	1.22	0.96; 1.56	.115	1.21	0.95; 1.56	.120
Italy	0.12	0.04; 0.19	.003	0.12	0.05; 0.20	.002	1.89	1.57; 2.27	<.001	1.90	1.58; 2.29	<.001
Spain	0.00	-0.06; 0.07	.958	-0.00	-0.07; 0.07	.993	1.49	1.28; 1.73	<.001	1.48	1.28; 1.73	<.001
Sweden	-0.06	-0.12; 0.00	.061	-0.05	-0.11; 0.01	.095	2.33	2.00; 2.73	<.001	2.35	2.01; 2.74	<.001
Switzerland	-0.20	-0.26; -0.13	<.001	-0.20	-0.26; -0.13	<.001	2.39	2.01; 2.83	<.001	2.39	2.01; 2.84	<.001
*Ability to make ends meet (ref: Easily)*												
Fairly easily	0.11	0.08; 0.14	<.001	0.11	0.08; 0.15	0.001	0.85	0.78; 0.92	<.001	0.85	0.79; 0.92	<.001
With some difficulty	0.12	0.07; 0.17	<.001	0.13	0.08; 0.17	<.001	0.75	0.67; 0.83	<.001	0.75	0.68; 0.84	<.001
With great difficulty	0.17	0.10; 0.25	<.001	0.18	0.10; 0.25	<.001	0.63	0.53; 0.74	<.001	0.63	0.53; 0.74	<.001
*Education (ref: Primary)*												
Secondary	-0.13	-0.17; -0.09	<.001	-0.13	-0.17; -0.09	<.001	1.17	1.07; 1.29	.001	1.17	1.07; 1.28	.001
Tertiary	-0.26	-0.31; -0.20	<.001	-0.26	-0.31; -0.21	<.001	1.29	1.14; 1.45	<.001	1.28	1.13; 1.44	<.001
*Occupation status (ref: High skill)*												
Low skill	0.08	0.05; 0.11	<.001	0.10	0.07; 0.13	<.001	0.95	0.87; 1.02	.166	0.96	0.88; 1.04	.281
Never worked	0.03	-0.04; 0.10	.471	0.08	0.01; 0.15	.035	0.65	0.55; 0.75	<.001	0.68	0.58; 0.80	<.001

*Note:* BMI: body mass index; PA: Physical activity status. Model 3a and Model 4a included interaction terms between gender and material deprivation on BMI (Model 3a) and between gender and material deprivation and BMI and gender on physical activity (Model 4a). Unstandardized b coefficients and odds-ratios (OR) and their 95% confidence intervals (95% CI) are reported.

**Fig. 1:**
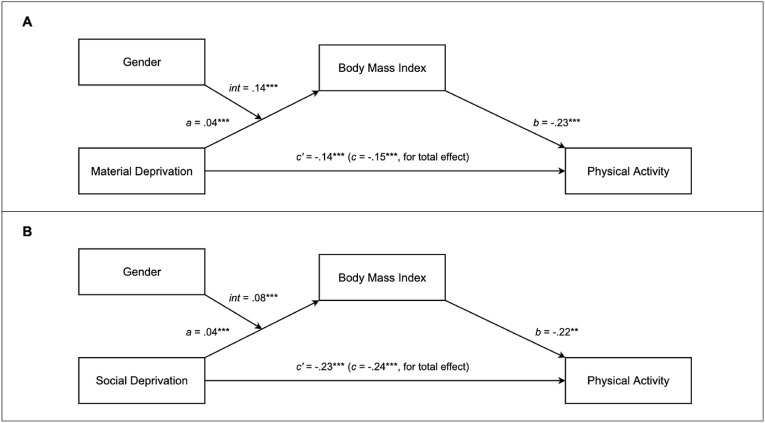
Moderated mediation models with material deprivation (A), social deprivation (B). *Note.* ***: p < .001. Int: Moderating effect of gender on the *a* path. All paths are expressed as beta coefficients.

**Fig. 2:**
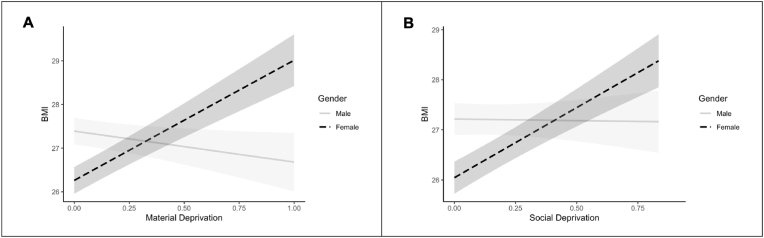
Simples slopes for the interaction of material deprivation (A) and social deprivation with gender on BMI.

Results of the moderated mediation analyses showed that the indirect effect of material deprivation on physical activity through BMI was moderated by gender (index of the moderated indirect effect = 0.0058, 95% CI = [0.0041; 0.0077], *p* < .001) (Fig. 1A). Relative to men (index of the indirect effect = 0.0013, 95% CI = [0.0001; 0.0026], *p* = .040), the indirect effect was stronger among women (index of the indirect effect = -0.0045, 95% CI = [-0.0060; -0.0032], *p* < .001). This stronger indirect effect was accounted by the significant moderating effect of gender on the association between material deprivation and BMI (*a* path) (Model 3a: b = 0.14, 95% CI = [0.11; 0.17], *p* < .001). Simple slope analysis revealed that the association of material deprivation with BMI was stronger among women (b = 0.11, 95% CI = [0.09; 0.13], *p* < .001), relative to men (b = -0.03, 95% CI = [-0.05; -0.01], *p* = .030) (Fig. 2A). Gender did not significantly moderate the strength of the association between BMI and physical activity (Model 4a, *b* path) (*p* = .230). Among women, BMI mediated around 18% of the association between material deprivation and physical activity, against 4% among men.

### 2.2 Social deprivation

Results of the simple mediation model showed a significant indirect effect of social deprivation on physical activity through BMI (index of the indirect effect = -0.0019, 95% CI = [-0.0027; -0.0011], *p* < .001) (Table 3). Specifically, social deprivation was positively associated with BMI (Model 1b: b = 0.05, 95% CI = [0.03; 0.06], *p* < .001) and, in turn, higher BMI was associated with lower odds of being physically active (vs inactive) (Model 2b: b = -0.21, 95% CI = [-0.24 -0.18], OR = 0.81, 95% CI = [0.79; 0.84], *p* < .001). The direct effect of social deprivation on physical activity remained significant after adjustment for BMI (Model 2b: b = -0.23, 95% CI = [-0.27; -0.19], OR = 0.79, 95% CI = [0.77; 0.82], *p* < .001). In sum, BMI partly mediated the association of social deprivation with physical activity (total effect *c* = -0.24, proportion of mediated effect = 4%).

**Table 3:** Estimates for all predictors in the models including social deprivation.

Predictors	Model 1b (BMI as outcome)			Model 3b (BMI as outcome)			Model 2b (PA as outcome)			Model 4b (PA as outcome)		
	b	95% CI	*p*	b	95% CI	*p*	OR	95% CI	*p*	OR	95% CI	*p*
Intercept	-0.01	-0.08; 0.05	.665	-0.02	-0.09; 0.04	.528	2.01	1.73; 2.34	<.001	2.01	1.73; 2.33	<.001
Social deprivation	0.05	0.03; 0.06	<.001	0.04	0.02; 0.06	<.001	0.79	0.77; 0.82	<.001	0.79	0.76; 0.82	<.001
BMI	–	–	–				0.81	0.79; 0.84	<.001	0.80	0.78; 0.83	<.001
Gender (ref: men)	–	–	–	-0.15	-0.17; -0.12	<.001	–	–	–	0.89	0.83; 0.95	<.001
Social deprivation × gender	–	–	–	0.08	0.06; 0.11	<.001	–	–	–	1.06	0.99; 1.13	.107
BMI × gender	–	–	–	–	–	–	–	–	–	1.04	0.97; 1.11	.238
Age	-0.07	-0.09; -0.06	<.001	-0.08	-0.09; -0.06	<.001	0.57	0.55; 0.60	<.001	0.57	0.55; 0.59	<.001
*Country (ref: Belgium)*												
Austria	0.13	0.06; 0.20	<.001	0.14	0.07; 0.21	<.001	1.72	1.45; 2.04	<.001	1.74	1.47; 2.06	<.001
Czech Republic	0.28	0.22; 0.35	<.001	0.30	0.24; 0.36	<.001	1.38	1.19; 1.60	<.001	1.40	1.21; 1.62	<.001
Denmark	0.04	-0.11; 0.02	.168	0.04	-0.10; 0.02	.230	2.01	1.71; 2.38	<.001	2.02	1.72; 2.39	<.001
Estonia	0.23	0.16; 0.29	<.001	0.25	0.18; 0.31	<.001	1.36	1.18; 1.58	<.001	1.38	1.19; 1.60	<.001
France	-0.09	-0.16; -0.03	.004	-0.09	-0.15; -0.03	.006	1.14	0.98; 1.32	.083	1.14	0.99; 1.32	.075
Germany	0.14	0.08; 0.20	<.001	0.14	0.08; 0.20	<.001	1.43	1.25; 1.65	<.001	1.44	1.25; 1.65	<.001
Israël	0.13	0.01; 0.24	.028	0.13	0.02; 0.24	.019	0.87	0.68; 1.11	.249	0.87	0.68; 1.11	.268
Luxembourg	-0.20	-0.27; -0.14	<.001	-0.21	-0.28; -0.15	<.001	0.80	0.69; 0.93	.003	0.79	0.69; 0.92	.002
Slovenia	0.10	-0.01; 0.20	.068	0.09	-0.01; 0.19	.082	1.22	0.96; 1.57	.107	1.22	0.96; 1.57	.111
Italy	0.14	0.06; 0.21	.001	0.14	0.07; 0.22	<.001	1.74	1.45; 2.09	<.001	1.75	1.46; 2.11	<.001
Spain	0.01	-0.05; 0.08	.707	0.00	-0.06; 0.07	.897	1.46	1.26; 1.70	<.001	1.45	1.25; 1.69	<.001
Sweden	-0.05	-0.11;-0.02	.142	-0.04	-0.10; -0.02	.199	2.23	1.91; 2.61	<.001	2.24	1.92; 2.62	<.001
Switzerland	-0.18	-0.25; -0.12	<.001	-0.18	-0.25; -0.12	<.001	2.26	1.90; 2.68	<.001	2.26	1.90; 2.68	<.001
*Ability to make ends meet (ref: Easily)*												
Fairly easily	0.12	0.08; 0.15	<.001	0.12	0.09; 0.15	<.001	0.84	0.78; 0.91	<.001	0.85	0.78; 0.92	<.001
With somedifficulty	0.16	0.12; 0.20	<.001	0.17	0.13; 0.21	<.001	0.71	0.65; 0.79	<.001	0.72	0.65; 0.79	<.001
With great difficulty	0.25	0.19; 0.31	<.001	0.25	0.19; 0.31	<.001	0.58	0.50; 0.67	<.001	0.58	0.50; 0.67	<.001
*Education (ref: Primary)*												
Secondary	-0.12	-0.16; -0.08	<.001	-0.13	-0.17; -0.09	<.001	1.14	1.04; 1.25	.004	1.14	1.04; 1.25	.005
Tertiary	-0.25	-0.30; -0.20	<.001	-0.25	-0.30; -0.20	<.001	1.23	1.09; 1.39	.001	1.23	1.09; 1.38	.001
*Occupation status (ref: High skill)*												
Low skill	0.08	0.05; 0.11	<.001	0.09	0.06; 0.12	<.001	0.97	0.89; 1.05	.411	0.98	0.90; 1.06	.552
Never worked	0.02	-0.05; 0.09	.639	0.07	-0.00; -0.14	.051	0.68	0.58; 0.80	<.001	0.72	0.61; 0.84	<.001

*Note:* Model 3b and Model 4b included an interaction terms between gender and social deprivation on BMI (Model 3b), between gender and social deprivation and BMI and gender on physical activity (Model 4b). Unstandardized b coefficients and odds-ratios (OR) and their 95% confidence intervals (95% CI) are reported.

Results of the moderated mediation analyses showed that the indirect effect of social deprivation on physical activity through BMI was moderated by gender (index of the moderated indirect effect = 0.0034, 95% CI = [0.0019; 0.0050], *p* < .001) (Fig. 1B). Relative to men (index of the indirect effect = 0.0001, 95% CI = [-0.0009; 0.0011], *p* = .860), the indirect effect was significant and stronger among women (index of the indirect effect = -0.0033, 95% CI = [-0.0045; -0.0022], *p* < .001). This stronger indirect effect was accounted by the significant moderating effect of gender on the association between social deprivation and BMI (*a* path) (Model 3b: b = 0.08, 95% CI = [0.06; 0.11], *p* < .001). Simple slope analysis revealed that the association of social deprivation with BMI was stronger among women (b = 0.08, 95% CI = [0.06; 0.10], *p* < .001), relative to men (b = -0.00, 95% CI = [-0.02; 0.02], *p* = .870) (Fig. 2B). Gender did not significantly moderate the strength of the association between BMI and physical activity (Model 4b, *b* path) (*p* = .238). Among women, BMI mediated around 7% of the association between social deprivation and physical activity, against 2% among men.

### 2.3 Robustness analyses

Results remained consistent in models further adjusting for ADL and IADL as potential confounding variables (Tables S2–S5).

Wave of measurement was neither associated with BMI nor with physical activity, with results remaining consistent with those observed in previous analyses (Tables S6 and S7).

## 3 Discussion

### 3.1 Main findings

Findings from this European large-scale study showed that, among adults over 50 years of age, BMI partly mediated the association between deprivation and physical activity. Moreover, this mediating pathway was moderated by gender, with the indirect effect of deprivation on physical activity through BMI being stronger among women, relative to men. This stronger mediating effect was explained by a more pronounced association between deprivation and BMI among women vs men. Overall, these findings extend our understanding of the gender-patterned pathways through which deprivation relates to physical activity.

### 3.2 Comparison with previous studies

Our results align with past literature demonstrating that deprived adults report lower levels of physical activity in Western countries, including among older populations (O'Donoghue et al., 2020). Both material and social deprivation were negatively associated with physical activity, with this association being descriptively and slightly stronger for social (vs material) deprivation. In search of underlying mechanisms, as hypothesized (H1), our results suggested that the relationship between deprivation and physical activity was partly explained by BMI, after adjustment for age, country of residence, education and ability to make ends meet and occupation. As such, perceiving a high level of deprivation may increase the risk of weight gain among older adults, which may in turn impede the adoption of physically active behaviors. A mechanism that may ultimately create a vicious circle or an accumulation of disadvantages (Dannefer, 2003) on health status.

Studies conducted among adolescents also evidenced this mediating pathway, though they assumed a reversed causal pathway. They showed that the association of deprivation and BMI was attenuated (or even non-significant), when controlling for physical activity (Clennin et al., 2020; Nevill et al., 2016). Here, we examined the association from BMI to physical activity as recent evidence rather leads us to postulate that BMI is likely to precede physical activity (Sagelv et al., 2021; Skrede et al., 2021). Indeed, these two studies testing the bi-directional association between physical activity and BMI suggested that BMI was likely to precede physical activity, rather than the opposite (Sagelv et al., 2021; Skrede et al., 2021). Moreover, it should be noted that the effect of physical activity on weight loss remains debated (Pontzer, 2015), especially among older adults among whom physical activity rather relates to weight maintenance (Stephen & Janssen, 2010). While several mechanisms have been put forth to explain the detrimental effects of BMI on physical activity, spanning from physiological (e.g., higher energy expenditure, mechanical workloads) (Lafortuna et al., 2008), socio-cultural (e.g., stronger stigmatization) (Vartanian & Shaprow, 2008) to psychological pathways (e.g., more negative affective responses or lower self-efficacy) (Dikareva et al., 2016; Ekkekakis & Lind, 2006), future research remains needed to disentangle the potential bi-directional relationships between BMI and physical activity in disadvantaged populations.

As predicted (H2), the mediating pathway between deprivation and physical activity through BMI was moderated by participants’ gender. Specifically, we found consistent evidence for a moderating effect on the first component of the mediating pathway (i.e., from deprivation to BMI). In line with previous literature (Bann et al., 2017; Matheson et al., 2008; Shaw et al., 2014), relative to men, the association of deprivation with BMI was stronger among women. Although remaining speculative, several explanations can be put forth to explain this exacerbated relationship. First, from a sociocultural lens, it has been proposed that women reporting a high level of deprivation may be less likely to experience injunctions to thinness than women living in a more privileged milieu (McLaren & Kuh, 2004; Swami et al., 2010), especially at older age (Hajek & König, 2018). In turn, this weaker cultural pressure may expose deprived women to increases in BMI across aging (von Lengerke & Mielck, 2012). Conversely, irrespectively from socio-economic circumstances, men are exposed to weaker injunctions to thinness (McLaren & Kuh, 2004; Swami et al., 2010). From a social perspective, being a woman (vs a man) and experiencing a high deprivation increases odds of isolation (Nicolaisen & Thorsen, 2014), which could in turn foster weight gains due to the absence of social support (Kiernan et al., 2012). Finally, at the behavioral level, compared to men or to privileged women, women living in a disadvantaged environment are at higher risk to report a greater tendency to unhealthy food behaviors (e.g., skipping meal, binge eating) (Jeffery & French, 1996; Reagan & Hersch, 2005), which are related to a higher BMI (French et al., 2012). Again, these explanations would deserve to be tested by future studies, with the aim of shedding light on the mechanisms behind the exacerbated association between deprivation and BMI among older women.

Importantly, we found evidence for a partial mediation of the association between deprivation and physical activity through BMI at older age. Moreover, the proportion of mediated effects ranged from 4% to 8% for material and social deprivation respectively − though this proportion raised up to 18% among women. Although the magnitude of these effect sizes should be interpreted as non-trivial in light of the context of the study and may be of public health relevance (i.e., a weaker exposure to deprivation may lead to an increase in physical activity in the global population), this suggests that other factors may conjointly underlie the association between deprivation and physical activity, especially among men. While the role of environmental (e.g., access to facilities, walkability, adverse neighborhood conditions, air pollution) (Chaparro et al., 2018; Hillsdon et al., 2007; Li et al., 2005), cultural (e.g., class habitus) (Bourdieu, 1979; Cockerham et al., 2020; Pampel et al., 2010), or cognitive factors (e.g., motivations, cognitive functions) (Cheval et al., 2019; Hagger & Hamilton, 2020) was already identified, it seems critical to broaden our understanding of the gender-patterned mechanisms through which deprivation relates to physical activity. Additionally, other patterns of associations between deprivation and BMI could underlie their relative effect on physical activity. Particularly, future research could investigate the potential interactive effect of deprivation and BMI on physical activity: i.e., could the effect of deprivation on physical activity be stronger among individuals with obesity (relative to normal weight), such that the effect of socio-economic disadvantages would be exacerbated among individuals also suffering from a detrimental health condition?

### 3.3 Strengths and limitations

Among the main strengths of this study are the reliance on different indicators of deprivation, the large size of the sample, the time lag between variables, as well as the complementary of statistical approaches. However, this study has several limitations that should be acknowledged. First, we cannot rule out the possibility that BMI and physical activity might be bi-directionally associated. In the same line, a higher BMI may be related to a greater deprivation (Howe et al., 2020). Accordingly, future research remains needed before drawing any conclusion on the causality between observed variables. Second, the self-reported measure of physical activity lacks granularity and may have led to an overestimation of physical activity levels (Lee et al., 2011). BMI was also self-reported, which may have led to inaccuracies and triggered social desirability bias (Gorber et al., 2007). Other measures of participants' adiposity status (e.g., waist circumference, fat mass) would have been interesting to consider in relation to deprivation and physical activity (Müller et al., 2012). Similarly, deprivation was measured at the individual level, which is subject to social desirability and information biases, in comparison with area-based measures (i.e., measures based on participants’ postcode and aggregating objective indicators in the local area, such as employment rate). Third, the present study did not allow to disentangle leisure time and occupational physical activity, two contexts in which physical activity levels may differ depending on deprivation. Fourth, regarding inclusion criteria (i.e., having complete measures on three timepoints), a selection bias may have occurred, thereby reducing the representativeness of our sample (see Table S1 for a comparison of characteristics from all Wave 5 participants and of participants included in the analyses). In particular, participants included in the present analyses reported slightly lower scores on functional dependence in (instrumental) activities of daily life (Table S1) than all participants included in Wave 5, which may indicate an over-representation of participants with a good health status. Moreover, as the recruitment procedure of SHARE occurred at age 50 years and older, respondents may overall be more likely of better health than non-respondents, due to a survivor bias. This represents another risk for selection bias and inherently limits the generalization of the current findings to younger individuals.

## 4 Conclusions

Overall, this study showed, among older adults, that (1) BMI partly mediated the association between deprivation and physical activity and (2) that this mediating pathway was more pronounced among women (vs men) because of a stronger effect of deprivation on BMI among women. We hope that these findings will encourage future research in considering how deprivation and gender may interact to explain health behaviors, such as physical activity. Finally, public health authorities should pay a special attention in promoting physical activity among the most deprived older adults, and especially women, as well as addressing the specific barriers which refrain them from engaging in healthy lifestyles.

## Ethical statement

Declarations of interest: none.

## Data Availability

The authors do not have permission to share data.
