# Supplementary material for: Women carry the weight of deprivation on physical inactivity: Moderated mediation analyses in a European sample of adults over 50 Years of age
Source: SSM Popul Health. 2022 Oct 23;20:101272. doi: 10.1016/j.ssmph.2022.101272 (PMC9641026; doi:10.1016/j.ssmph.2022.101272)
Supplement: Multimedia component 1 [file mmc1.docx]

**Women Carry the Weight of Deprivation on Physical Inactivity: Moderated Mediation Analyses in a European Sample of Adults over 50 Years of Age**

**Supplementary material**

**Figure S1.** Flow chart of included participants.

**Table S1.** Comparison of participants’ characteristics at baseline (Wave 5) and of participants who were included in the analyses.

**Table S2.** Estimates for all predictors in the models including material deprivation, and functional dependence in activities of daily living.

**Table S3.** Estimates for all predictors in the models including social deprivation, and functional dependence in activities of daily living.

**Table S4.** Estimates for all predictors in the models including material deprivation, and functional dependence in instrumental activities of daily living.

**Table S5.** Estimates for all predictors in the models including social deprivation, and functional dependence in instrumental activities of daily living.

**Table S6.** Estimates for all predictors in the models including material deprivation and wave of measurement as a confounding variable.

**Table S7.** Estimates for all predictors in the models including social deprivation and wave of measurement as a confounding variable.

**Figure S1.** Flow chart of included participants.


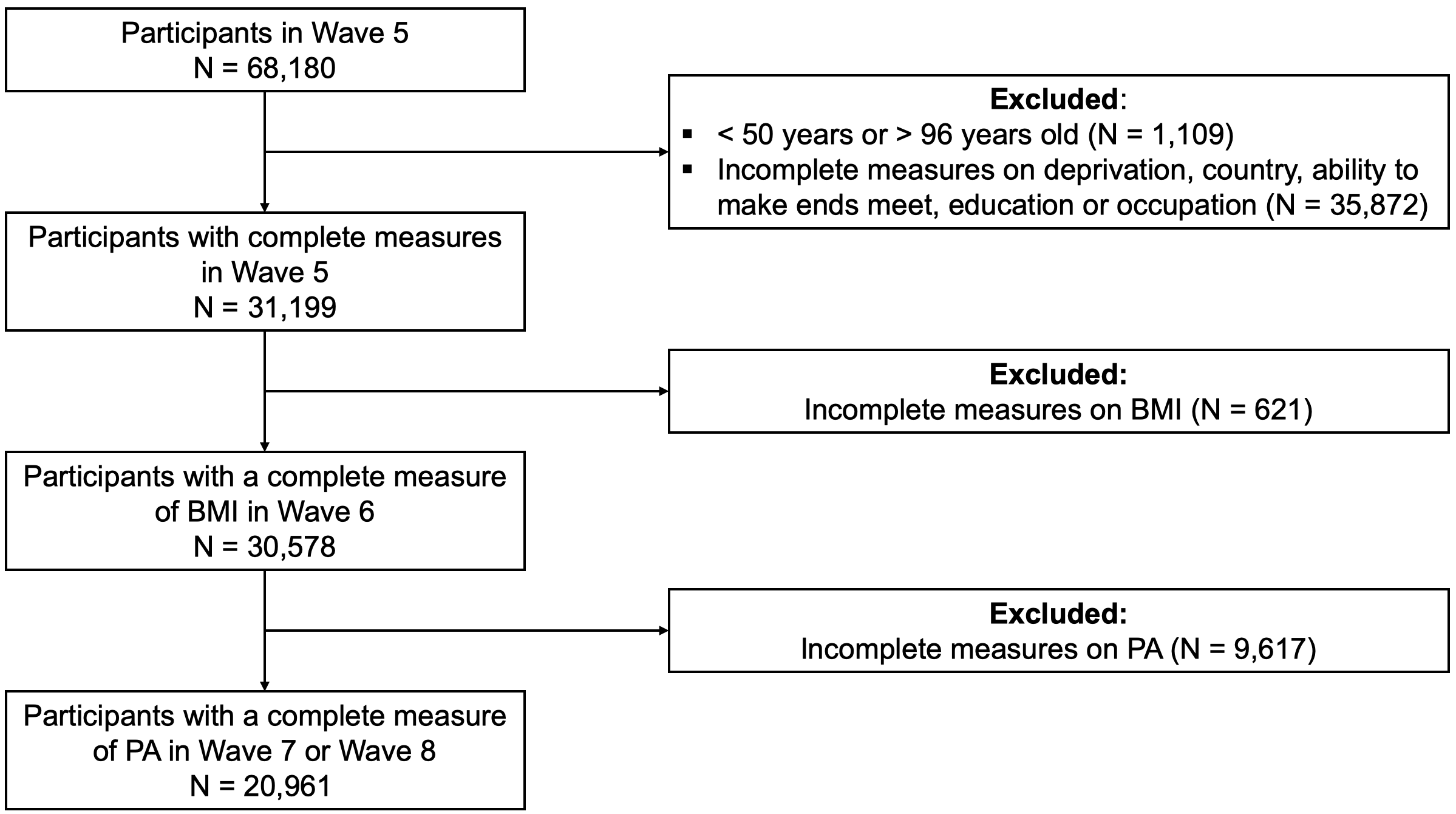


*Note.* BMI: Body mass index, PA: physical activity.

**Table S1.** Comparison of participants’ characteristics at baseline (Wave 5) and of participants who were included in the analyses.

|  | | **All participants at baseline**  **N = 35,675** | | | **Participants included**  **N = 20,961** | |
| --- | --- | --- | --- | --- | --- | --- |
|  | **N** | | **%** | **N** | | **%** |
| **Physical activity** |  | |  |  | |  |
| Physically active (N, %) | 26,498 | | 74% | 14,760 | | 70% |
| Physically inactive (N, %) | 9,177 | | 26% | 6,201 | | 30% |
| **Material deprivation** (Mean, SD) | 0.13 | | 0.19 | 0.12 | | 0.18 |
| **Social deprivation** (Mean, SD) | 0.17 | | 0.13 | 0.16 | | 0.13 |
| **BMI** (Mean, SD) | 26.9 | | 4.67 | 27.00 | | 4.60 |
| **Gender** (N, % of women) | 20,026 | | 56% | 11,898 | | 57% |
| **Age** (Mean, SD) | 66.09 | | 9.49 | 67.89 | | 8.76 |
| **Country** (N, %) |  | |  |  | |  |
| Austria | 2,600 | | 10% | 1197 | | 6% |
| Belgium | 3,437 | | 7% | 1914 | | 9% |
| Czech Republic | 2,813 | | 8% | 1787 | | 9% |
| Denmark | 2,458 | | 7% | 1720 | | 8% |
| Estonia | 3,095 | | 9% | 1802 | | 9% |
| France | 2,550 | | 7% | 1654 | | 8% |
| Germany | 3,107 | | 9% | 2308 | | 11% |
| Israël | 832 | | 2% | 347 | | 2% |
| Italy | 3,131 | | 9% | 1823 | | 9% |
| Luxembourg | 684 | | 2% | 424 | | 2% |
| Netherlands | 1,283 | | 4% | - | | - |
| Slovenia | 1,478 | | 4% | 952 | | 5% |
| Spain | 3,494 | | 10% | 1670 | | 8% |
| Sweden | 2,745 | | 8% | 1897 | | 9% |
| Switzerland | 1,968 | | 10% | 1466 | | 7% |
| **Education (N, %)** |  | |  |  | |  |
| Primary school | 7,125 | | 20% | 3868 | | 19% |
| Secondary school | 19,982 | | 56% | 11814 | | 56% |
| Tertiary school | 8568 | | 24% | 5279 | | 25% |
| **Ability to make ends (N, %)** |  | |  |  | |  |
| Fairly easily | 10,864 | | 31% | 6344 | | 30% |
| Easily | 15,797 | | 44% | 9545 | | 46% |
| With some difficulty | 6,600 | | 19% | 3779 | | 18% |
| With great difficulty | 2,414 | | 6% | 1293 | | 6% |
| **Occupation status (N, %)** |  | |  |  | |  |
| High skill | 12,072 | | 34% | 7348 | | 35% |
| Low skill | 21,823 | | 61% | 12623 | | 60% |
| Never worked | 1,780 | | 5% | 990 | | 5% |
| **ADL** (Mean, SD) | 0.18 | | 0.70 | 0.14 | | 0.61 |
| **IADL** (Mean, SD) | 0.27 | | 0.89 | 0.21 | | 0.74 |

*Note.* SD: Standard-deviation. Participants at baseline were included if they reported complete measures in Wave 5 on the variables listed in this table. To be included in the study, participants had to be between 50 years and 96 years old and had completed measures of deprivation in Wave 5, of BMI in Wave 6 and of physical activity in Wave 7 or in Wave 8.

ADL: functional dependence in activities of daily living at baseline (Wave 5), was expressed as a continuous score ranging from 0 to 6 and was assessed in reference of six ADL (dressing, walking, bathing, eating, getting in or out of bed, and using the toilet). IADL: functional dependence in instrumental activities of daily living at baseline (Wave 5), was expressed as a continuous score ranging from 0 to 7 and was assessed in reference of seven IADL (using a map, preparing a hot meal, shopping for groceries, making telephone calls, taking medication, gardening or doing housework, and managing money).

**Table S2**. Estimates for all predictors in the models including material deprivation, and functional dependence in activities of daily living (ADL).

|  | **Model 1a (BMI as outcome)** | | | **Model 3a (BMI as outcome)** | | | **Model 2a (PA as outcome)** | | | **Model 4a (PA as outcome)** | | | |
| --- | --- | --- | --- | --- | --- | --- | --- | --- | --- | --- | --- | --- | --- |
| **Predictors** | **b** | **95% CI** | ***p*** | **b** | **95% CI** | ***p*** | **OR** | **95% CI** | ***p*** | **OR** | **95% CI** | | ***p*** |
| Intercept | -0.01 | -0.07 – 0.06 | 0.816 | -0.02 | -0.08 – 0.05 | 0.605 | 2.08 | 1.78 – 2.42 | <0.001 | 2.07 | 1.78 – 2.42 | <0.001 | |
| Material deprivation | 0.05 | 0.03 – 0.07 | <0.001 | 0.04 | 0.02 – 0.06 | <0.001 | 0.90 | 0.86 – 0.94 | <0.001 | 0.89 | 0.85 – 0.93 | <0.001 | |
| BMI | - | - | - |  |  |  | 0.82 | 0.80 – 0.85 | <0.001 | 0.81 | 0.79 – 0.84 | <0.001 | |
| Gender (ref : men) | - | - | - | -0.14 | -0.17 – -0.12 | <0.001 | - | - | - | 0.88 | 0.82 – 0.94 | <0.001 | |
| Material deprivation × gender | - | - | - | 0.14 | 0.11 – 0.17 | <0.001 | - | - | - | 1.06 | 0.99 – 1.13 | 0.075 | |
| BMI × gender | - | - | - | - | - | - | - | - | - | 1.04 | 0.97 – 1.11 | 0.268 | |
| Age | -0.07 | -0.09 – -0.06 | <0.001 | -0.08 | -0.10 – -0.06 | <0.001 | 0.57 | 0.55 – 0.59 | <0.001 | 0.57 | 0.54 – 0.59 | <0.001 | |
| *Country (ref: Belgium)* | | | | | | | | | | | | | |
| Austria | 0.12 | 0.05 – 0.19 | 0.001 | 0.13 | 0.06 – 0.20 | <0.001 | 1.79 | 1.51 – 2.12 | <0.001 | 1.81 | 1.53 – 2.15 | <0.001 | |
| Czech Republic | 0.30 | 0.24 – 0.36 | <0.001 | 0.31 | 0.25 – 0.38 | <0.001 | 1.22 | 1.06 – 1.41 | 0.007 | 1.24 | 1.07 – 1.43 | 0.004 | |
| Denmark | -0.06 | -0.12 – 0.00 | 0.062 | -0.05 | -0.12 – 0.01 | 0.092 | 2.17 | 1.84 – 2.57 | <0.001 | 2.19 | 1.85 – 2.58 | <0.001 | |
| Estonia | 0.19 | 0.13 – 0.26 | <0.001 | 0.21 | 0.14 – 0.27 | <0.001 | 1.43 | 1.23 – 1.67 | <0.001 | 1.45 | 1.24 – 1.69 | <0.001 | |
| France | -0.09 | -0.16 – -0.03 | 0.005 | -0.09 | -0.15 – -0.02 | 0.008 | 1.10 | 0.95 – 1.28 | 0.198 | 1.11 | 0.96 – 1.28 | 0.176 | |
| Germany | 0.13 | 0.07 – 0.19 | <0.001 | 0.13 | 0.07 – 0.19 | <0.001 | 1.47 | 1.28 – 1.70 | <0.001 | 1.47 | 1.28 – 1.70 | <0.001 | |
| Israël | 0.13 | 0.02 – 0.24 | 0.024 | 0.14 | 0.03 – 0.25 | 0.014 | 0.81 | 0.63 – 1.03 | 0.086 | 0.81 | 0.63 – 1.04 | 0.096 | |
| Luxembourg | -0.19 | -0.26 – -0.13 | <0.001 | -0.20 | -0.26 – -0.13 | <0.001 | 0.74 | 0.64 – 0.86 | <0.001 | 0.74 | 0.64 – 0.85 | <0.001 | |
| Slovenia | 0.09 | -0.01 – 0.19 | 0.086 | 0.08 | -0.02 – 0.19 | 0.104 | 1.25 | 0.98 – 1.61 | 0.078 | 1.25 | 0.98 – 1.60 | 0.082 | |
| Italy | 0.13 | 0.05 – 0.21 | 0.001 | 0.14 | 0.06 – 0.21 | <0.001 | 1.79 | 1.49 – 2.15 | <0.001 | 1.80 | 1.50 – 2.17 | <0.001 | |
| Spain | 0.01 | -0.06 – 0.07 | 0.839 | 0.00 | -0.06 – 0.07 | 0.889 | 1.46 | 1.25 – 1.70 | <0.001 | 1.45 | 1.25 – 1.69 | <0.001 | |
| Sweden | -0.05 | -0.12 – 0.01 | 0.089 | -0.05 | -0.11 – 0.01 | 0.134 | 2.29 | 1.96 – 2.68 | <0.001 | 2.31 | 1.97 – 2.70 | <0.001 | |
| Switzerland | -0.19 | -0.25 – -0.12 | <0.001 | -0.18 | -0.25 – -0.12 | <0.001 | 2.29 | 1.93 – 2.72 | <0.001 | 2.29 | 1.93 – 2.72 | <0.001 | |
| ADL | 0.11 | 0.09 – 0.13 | <0.001 | 0.11 | 0.09 – 0.14 | <0.001 | 0.63 | 0.59 – 0.67 | <0.001 | 0.63 | 0.59 – 0.67 | <0.001 | |
| *Ability to make ends (ref: Easily)* | | | | | | | | | | | | | |
| Fairly easily | 0.11 | 0.07 – 0.14 | <0.001 | 0.11 | 0.08 – 0.14 | <0.001 | 0.86 | 0.79 – 0.93 | <0.001 | 0.86 | 0.79 – 0.93 | <0.001 | |
| With some  difficulty | 0.11 | 0.07 – 0.16 | <0.001 | 0.12 | 0.08 – 0.17 | <0.001 | 0.77 | 0.69 – 0.85 | <0.001 | 0.77 | 0.69 – 0.86 | <0.001 | |
| With great difficulty | 0.15 | 0.08 – 0.23 | <0.001 | 0.16 | 0.08 – 0.23 | <0.001 | 0.67 | 0.56 – 0.79 | <0.001 | 0.67 | 0.57 – 0.79 | <0.001 | |
| *Education (ref: Primary)* | | | | | | | | | | | | | |
| Secondary | -0.12 | -0.17 – -0.08 | <0.001 | -0.13 | -0.17 – -0.09 | <0.001 | 1.18 | 1.07 – 1.29 | 0.001 | 1.17 | 1.07 – 1.28 | 0.001 | |
| Tertiary | -0.25 | -0.30 – -0.20 | <0.001 | -0.26 | -0.31 – -0.21 | <0.001 | 1.28 | 1.14 – 1.44 | <0.001 | 1.27 | 1.13 – 1.43 | <0.001 | |
| *Occupation status (ref: High skill)* | | | | | | | | | | | | | |
| Low skill | 0.08 | 0.05 – 0.11 | <0.001 | 0.10 | 0.06 – 0.13 | <0.001 | 0.95 | 0.88 – 1.03 | 0.252 | 0.97 | 0.89 – 1.05 | 0.391 | |
| Never worked | 0.02 | -0.05 – 0.09 | 0.655 | 0.07 | -0.00 – 0.14 | 0.064 | 0.66 | 0.56 – 0.77 | <0.001 | 0.69 | 0.59 – 0.81 | <0.001 | |

*Note:* Model 3a included an interaction term between gender and social deprivation on BMI, Model 4a included interaction terms between gender and material deprivation and gender and BMI on physical activity. Unstandardized b coefficients and odds-ratios (OR) and their 95% confidence intervals (95% CI) are reported. ADL: functional dependence in activities of daily living at baseline (Wave 5), was expressed as a continuous score ranging from 0 to 6 and was assessed in reference of six ADL (dressing, walking, bathing, eating, getting in or out of bed, and using the toilet).

**Table S3**. Estimates for all predictors in the models including social deprivation, and functional dependence in activities of daily living (ADL).

|  | **Model 1b (BMI as outcome)** | | | **Model 3b (BMI as outcome)** | | | **Model 2b (PA as outcome)** | | | **Model 4b (PA as outcome)** | | | |  |  |  |  |  |  |  |  |
| --- | --- | --- | --- | --- | --- | --- | --- | --- | --- | --- | --- | --- | --- | --- | --- | --- | --- | --- | --- | --- | --- |
| **Predictors** | **b** | **95% CI** | ***p*** | **b** | **95% CI** | ***p*** | **OR** | **95% CI** | ***p*** | **OR** | **95% CI** | | ***p*** |  |  |  |  |  |  |  |  |
| Intercept | -0.03 | -0.10 – 0.03 | 0.355 | -0.04 | -0.10 – 0.03 | 0.263 | 2.13 | 1.83 – 2.48 | <0.001 | 2.12 | 1.83 – 2.48 | <0.001 | |  |  |  |  |  |  |  |  |
| Social deprivation | 0.03 | 0.02 – 0.05 | <0.001 | 0.03 | 0.01 – 0.04 | 0.001 | 0.83 | 0.80 – 0.86 | <0.001 | 0.83 | 0.80 – 0.86 | <0.001 | |  |  |  |  |  |  |  |  |
| BMI |  |  |  |  |  |  | 0.82 | 0.80 – 0.85 | <0.001 | 0.81 | 0.79 – 0.84 | <0.001 | |  |  |  |  |  |  |  |  |
| Gender (ref : men) | - | - | - | -0.15 | -0.17 – -0.12 | <0.001 | - | - | - | 0.89 | 0.83 – 0.95 | <0.001 | |  |  |  |  |  |  |  |  |
| Social deprivation × gender | - | - | - | 0.08 | 0.06 – 0.11 | <0.001 | - | - | - | 1.05 | 0.98 – 1.12 | 0.157 | |  |  |  |  |  |  |  |  |
| BMI × gender | - | - | - | - | - | - | - | - | - | 1.04 | 0.97 – 1.11 | 0.265 | |  |  |  |  |  |  |  |  |
| Age | -0.08 | -0.09 – -0.06 | <0.001 | -0.08 | -0.10 – -0.07 | - | - | - | <0.001 | 0.58 | 0.56 – 0.60 | <0.001 | |  |  |  |  |  |  |  |  |
| *Country (ref: Belgium)* | | | | | | | | | | | | | |  |  |  |  |  | - | - | - |
| Austria | 0.13 | 0.06 – 0.20 | <0.001 | 0.14 | 0.07 – 0.21 | <0.001 | 1.70 | 1.44 – 2.02 | <0.001 | 1.72 | 1.45 – 2.04 | <0.001 | |  |  |  |  |  |  |  |  |
| Czech Republic | 0.30 | 0.23 – 0.36 | <0.001 | 0.31 | 0.25 – 0.37 | <0.001 | 1.32 | 1.14 – 1.53 | <0.001 | 1.33 | 1.15 – 1.54 | <0.001 | |  |  |  |  |  |  |  |  |
| Denmark | -0.05 | -0.11 – 0.02 | 0.157 | -0.04 | -0.10 – 0.02 | 0.214 | 2.04 | 1.73 – 2.40 | <0.001 | 2.05 | 1.74 – 2.42 | <0.001 | |  |  |  |  |  |  |  |  |
| Estonia | 0.23 | 0.16 – 0.29 | <0.001 | 0.25 | 0.18 – 0.31 | <0.001 | 1.36 | 1.17 – 1.58 | <0.001 | 1.38 | 1.19 – 1.60 | <0.001 | |  |  |  |  |  |  |  |  |
| France | -0.09 | -0.15 – -0.02 | 0.008 | -0.08 | -0.15 – -0.02 | 0.011 | 1.11 | 0.96 – 1.29 | 0.167 | 1.11 | 0.96 – 1.29 | 0.152 | |  |  |  |  |  |  |  |  |
| Germany | 0.14 | 0.08 – 0.20 | <0.001 | 0.14 | 0.08 – 0.20 | <0.001 | 1.43 | 1.24 – 1.65 | <0.001 | 1.43 | 1.25 – 1.65 | <0.001 | |  |  |  |  |  |  |  |  |
| Israël | 0.13 | 0.02 – 0.24 | 0.022 | 0.14 | 0.03 – 0.25 | 0.015 | 0.84 | 0.65 – 1.07 | 0.163 | 0.84 | 0.66 – 1.08 | 0.176 | |  |  |  |  |  |  |  |  |
| Luxembourg | -0.19 | -0.26 – -0.13 | <0.001 | -0.20 | -0.27 – -0.14 | <0.001 | 0.77 | 0.66 – 0.89 | <0.001 | 0.76 | 0.66 – 0.88 | <0.001 | |  |  |  |  |  |  |  |  |
| Slovenia | 0.09 | -0.01 – 0.19 | 0.081 | 0.09 | -0.02 – 0.19 | 0.097 | 1.25 | 0.98 – 1.61 | 0.077 | 1.25 | 0.98 – 1.61 | 0.080 | |  |  |  |  |  |  |  |  |
| Italy | 0.15 | 0.07 – 0.22 | <0.001 | 0.15 | 0.08 – 0.23 | <0.001 | 1.68 | 1.40 – 2.02 | <0.001 | 1.69 | 1.41 – 2.04 | <0.001 | |  |  |  |  |  |  |  |  |
| Spain | 0.02 | -0.05 – 0.08 | 0.616 | 0.01 | -0.06 – 0.07 | 0.802 | 1.43 | 1.23 – 1.67 | <0.001 | 1.42 | 1.22 – 1.66 | <0.001 | |  |  |  |  |  |  |  |  |
| Sweden | -0.04 | -0.11 – 0.02 | 0.164 | -0.04 | -0.10 – 0.02 | 0.226 | 2.21 | 1.89 – 2.59 | <0.001 | 2.22 | 1.90 – 2.60 | <0.001 | |  |  |  |  |  |  |  |  |
| Switzerland | -0.17 | -0.24 – -0.11 | <0.001 | -0.17 | -0.24 – -0.11 | <0.001 | 2.19 | 1.85 – 2.60 | <0.001 | 2.19 | 1.85 – 2.60 | <0.001 | |  |  |  |  |  |  |  |  |
| ADL | 0.11 | 0.09 – 0.13 | <0.001 | 0.11 | 0.09 – 0.13 | <0.001 | 0.65 | 0.62 – 0.69 | <0.001 | 0.65 | 0.62 – 0.69 | <0.001 | |  |  |  |  |  |  |  |  |
| *Ability to make ends (ref: Easily)* | | | | | | | | | | | | | |  |  |  |  |  |  |  |  |
| Fairly easily | 0.12 | 0.08 – 0.15 | <0.001 | 0.12 | 0.09 – 0.15 | <0.001 | 0.85 | 0.78 – 0.92 | <0.001 | 0.85 | 0.79 – 0.92 | <0.001 | |  |  |  |  |  |  |  |  |
| With some  difficulty | 0.15 | 0.11 – 0.19 | <0.001 | 0.16 | 0.12 – 0.20 | <0.001 | 0.73 | 0.66 – 0.81 | <0.001 | 0.74 | 0.67 – 0.81 | <0.001 | |  |  |  |  |  |  |  |  |
| With great difficulty | 0.23 | 0.17 – 0.29 | <0.001 | 0.23 | 0.17 – 0.29 | <0.001 | 0.61 | 0.53 – 0.71 | <0.001 | 0.62 | 0.53 – 0.71 | <0.001 | |  |  |  |  |  |  |  |  |
| *Education (ref: Primary)* | | | | | | | | | | | | | |  |  |  |  |  |  |  |  |
| Secondary | -0.12 | -0.16 – -0.08 | <0.001 | -0.13 | -0.17 – -0.09 | <0.001 | 1.15 | 1.05 – 1.27 | 0.003 | 1.15 | 1.05 – 1.26 | 0.003 | |  |  |  |  |  |  |  |  |
| Tertiary | -0.25 | -0.30 – -0.20 | <0.001 | -0.25 | -0.30 – -0.20 | <0.001 | 1.24 | 1.10 – 1.39 | <0.001 | 1.23 | 1.09 – 1.39 | 0.001 | |  |  |  |  |  |  |  |  |
| *Occupation status (ref: High skill)* | | | | | | | | | | | | | |  |  |  |  |  |  |  |  |
| Low skill | 0.08 | 0.05 – 0.11 | <0.001 | 0.09 | 0.06 – 0.12 | <0.001 | 0.97 | 0.90 – 1.05 | 0.462 | 0.98 | 0.90 – 1.06 | 0.606 | |  |  |  |  |  |  |  |  |
| Never worked | 0.01 | -0.06 – 0.08 | 0.757 | 0.07 | -0.01 – 0.14 | 0.071 | 0.69 | 0.58 – 0.80 | <0.001 | 0.72 | 0.61 – 0.84 | <0.001 | |  |  |  |  |  |  |  |  |

*Note:* Model 3b included an interaction term between gender and social deprivation on BMI, Model 4b included interaction terms between gender and material deprivation and gender and BMI on physical activity Unstandardized b coefficients and odds-ratios (OR) and their 95% confidence intervals (95% CI) are reported. ADL: functional dependence in activities of daily living at baseline (Wave 5), was expressed as a continuous score ranging from 0 to 6 and was assessed in reference of six ADL (dressing, walking, bathing, eating, getting in or out of bed, and using the toilet).

**Table S4**. Estimates for all predictors in the models including material deprivation, and functional dependence in instrumental activities of daily living (IADL).

|  | **Model 1a (BMI as outcome)** | | | **Model 3a (BMI as outcome)** | | | **Model 2a (PA as outcome)** | | | **Model 4a (PA as outcome)** | | | |
| --- | --- | --- | --- | --- | --- | --- | --- | --- | --- | --- | --- | --- | --- |
| Predictors | b | 95% CI | *p* | b | 95% CI | *p* | OR | 95% CI | *p* | OR | 95% CI | | *p* |
| Intercept | -0.00 | -0.07 – 0.06 | 0.965 | -0.01 | -0.08 – 0.05 | 0.729 | 2.21 | 1.89 – 2.58 | <0.001 | 2.20 | 1.88 – 2.57 | <0.001 | |
| Material deprivation | 0.05 | 0.03 – 0.07 | <0.001 | 0.04 | 0.02 – 0.06 | <0.001 | 0.91 | 0.87 – 0.95 | <0.001 | 0.91 | 0.87 – 0.95 | <0.001 | |
| BMI | - | - | - |  |  |  | 0.81 | 0.79 – 0.84 | <0.001 | 0.80 | 0.78 – 0.83 | <0.001 | |
| Gender (ref : men) | - | - | - | -0.15 | -0.17 – -0.12 | <0.001 | - | - | - | 0.90 | 0.85 – 0.97 | 0.003 | |
| Material deprivation × gender | - | - | - | 0.14 | 0.11 – 0.17 | <0.001 | - | - | - | 1.08 | 1.01 – 1.15 | 0.028 | |
| BMI × gender | - | - | - | - | - | - | - | - | - | 1.05 | 0.98 – 1.12 | 0.163 | |
| Age | -0.07 | -0.09 – -0.05 | <0.001 | -0.08 | -0.09 – -0.06 | <0.001 | 0.59 | 0.57 – 0.61 | <0.001 | 0.59 | 0.56 – 0.61 | <0.001 | |
| *Country (ref: Belgium)* | | | | | | | | | | | | | |
| Austria | 0.11 | 0.04 – 0.18 | 0.002 | 0.13 | 0.06 – 0.20 | <0.001 | 1.84 | 1.55 – 2.19 | <0.001 | 1.86 | 1.57 – 2.22 | <0.001 | |
| Czech Republic | 0.30 | 0.23 – 0.36 | <0.001 | 0.31 | 0.25 – 0.37 | <0.001 | 1.22 | 1.06 – 1.42 | 0.007 | 1.24 | 1.07 – 1.43 | 0.005 | |
| Denmark | -0.06 | -0.13 – 0.00 | 0.053 | -0.06 | -0.12 – 0.01 | 0.081 | 2.18 | 1.85 – 2.58 | <0.001 | 2.19 | 1.86 – 2.59 | <0.001 | |
| Estonia | 0.19 | 0.13 – 0.26 | <0.001 | 0.21 | 0.14 – 0.27 | <0.001 | 1.41 | 1.21 – 1.65 | <0.001 | 1.43 | 1.22 – 1.66 | <0.001 | |
| France | -0.09 | -0.16 – -0.03 | 0.004 | -0.09 | -0.15 – -0.02 | 0.007 | 1.07 | 0.93 – 1.25 | 0.344 | 1.08 | 0.93 – 1.25 | 0.313 | |
| Germany | 0.13 | 0.07 – 0.19 | <0.001 | 0.13 | 0.07 – 0.19 | <0.001 | 1.46 | 1.26 – 1.68 | <0.001 | 1.46 | 1.27 – 1.68 | <0.001 | |
| Israël | 0.12 | 0.01 – 0.23 | 0.038 | 0.13 | 0.02 – 0.24 | 0.024 | 0.89 | 0.69 – 1.15 | 0.374 | 0.90 | 0.70 – 1.16 | 0.405 | |
| Luxembourg | -0.19 | -0.26 – -0.13 | <0.001 | -0.20 | -0.26 – -0.13 | <0.001 | 0.70 | 0.60 – 0.81 | <0.001 | 0.69 | 0.60 – 0.80 | <0.001 | |
| Slovenia | 0.09 | -0.01 – 0.20 | 0.075 | 0.09 | -0.01 – 0.19 | 0.091 | 1.24 | 0.97 – 1.60 | 0.084 | 1.24 | 0.97 – 1.60 | 0.085 | |
| Italy | 0.12 | 0.04 – 0.20 | 0.002 | 0.13 | 0.05 – 0.21 | 0.001 | 1.76 | 1.46 – 2.12 | <0.001 | 1.77 | 1.47 – 2.13 | <0.001 | |
| Spain | 0.00 | -0.06 – 0.07 | 0.904 | 0.00 | -0.06 – 0.07 | 0.955 | 1.45 | 1.24 – 1.69 | <0.001 | 1.45 | 1.24 – 1.69 | <0.001 | |
| Sweden | -0.06 | -0.12 – 0.01 | 0.078 | -0.05 | -0.11 – 0.01 | 0.120 | 2.23 | 1.91 – 2.61 | <0.001 | 2.24 | 1.92 – 2.62 | <0.001 | |
| Switzerland | -0.19 | -0.26 – -0.13 | <0.001 | -0.19 | -0.26 – -0.12 | <0.001 | 2.25 | 1.89 – 2.67 | <0.001 | 2.25 | 1.90 – 2.68 | <0.001 | |
| IADL | 0.04 | 0.02 – 0.06 | <0.001 | 0.04 | 0.02 – 0.06 | <0.001 | 0.61 | 0.58 – 0.64 | <0.001 | 0.61 | 0.58 – 0.64 | <0.001 | |
| *Ability to make ends (ref: Easily)* | | | | | | | | | | | | | |
| Fairly easily | 0.11 | 0.07 – 0.14 | <0.001 | 0.11 | 0.08 – 0.15 | <0.001 | 0.85 | 0.79 – 0.93 | <0.001 | 0.86 | 0.79 – 0.93 | <0.001 | |
| With some  difficulty | 0.12 | 0.07 – 0.16 | <0.001 | 0.13 | 0.08 – 0.17 | <0.001 | 0.76 | 0.69 – 0.85 | <0.001 | 0.77 | 0.69 – 0.85 | <0.001 | |
| With great difficulty | 0.16 | 0.09 – 0.24 | <0.001 | 0.17 | 0.09 – 0.24 | <0.001 | 0.68 | 0.58 – 0.81 | <0.001 | 0.68 | 0.58 – 0.81 | <0.001 | |
| *Education (ref: Primary)* | | | | | | | | | | | | | |
| Secondary | -0.12 | -0.16 – -0.08 | <0.001 | -0.13 | -0.17 – -0.09 | <0.001 | 1.13 | 1.03 – 1.24 | 0.009 | 1.13 | 1.03 – 1.24 | 0.011 | |
| Tertiary | -0.25 | -0.30 – -0.20 | <0.001 | -0.26 | -0.31 – -0.21 | <0.001 | 1.23 | 1.09 – 1.38 | 0.001 | 1.22 | 1.08 – 1.37 | 0.001 | |
| *Occupation status (ref: High skill)* | | | | | | | | | | | | | |
| Low skill | 0.08 | 0.05 – 0.11 | <0.001 | 0.10 | 0.06 – 0.13 | <0.001 | 0.97 | 0.90 – 1.05 | 0.452 | 0.98 | 0.91 – 1.06 | 0.625 | |
| Never worked | 0.02 | -0.05 – 0.09 | 0.653 | 0.07 | -0.00 – 0.14 | 0.064 | 0.70 | 0.60 – 0.82 | <0.001 | 0.73 | 0.62 – 0.86 | <0.001 | |

*Note.* Model 3a included an interaction term between gender and social deprivation on BMI, Model 4a included interaction terms between gender and material deprivation and gender and BMI on physical activity. Unstandardized b coefficients and odds-ratios (OR) and their 95% confidence intervals (95% CI) are reported. IADL: functional dependence in instrumental activities of daily living at baseline (Wave 5), was expressed as a continuous score ranging from 0 to 7 and was assessed in reference of seven IADL (using a map, preparing a hot meal, shopping for groceries, making telephone calls, taking medication, gardening or doing housework, and managing money).

**Table S5**. Estimates for all predictors in the models including social deprivation, and functional dependence in instrumental activities of daily living (IADL).

|  | **Model 1b (BMI as outcome)** | | | **Model 3b (BMI as outcome)** | | | **Model 2a (PA as outcome)** | | | **Model 4b (PA as outcome)** | | | |
| --- | --- | --- | --- | --- | --- | --- | --- | --- | --- | --- | --- | --- | --- |
| **Predictors** | **b** | **95% CI** | ***p*** | **b** | **95% CI** | ***p*** | **OR** | **95% CI** | ***p*** | **OR** | **95% CI** | | ***p*** |
| Intercept | -0.02 | -0.09 – 0.04 | 0.483 | -0.03 | -0.10 – 0.03 | 0.362 | 2.24 | 1.92 – 2.61 | <0.001 | 2.23 | 1.92 – 2.60 | <0.001 | |
| Social deprivation | 0.04 | 0.02 – 0.06 | <0.001 | 0.03 | 0.02 – 0.05 | <0.001 | 0.85 | 0.82 – 0.88 | <0.001 | 0.84 | 0.81 – 0.88 | <0.001 | |
| BMI | - | - | - |  |  |  | 0.82 | 0.79 – 0.84 | <0.001 | 0.81 | 0.78 – 0.83 | <0.001 | |
| Gender (ref : men) | - | - | - | -0.15 | -0.18 – -0.12 | <0.001 | - | - | - | 0.91 | 0.85 – 0.97 | 0.006 | |
| Social deprivation × gender | - | - | - | 0.08 | 0.06 – 0.11 | <0.001 | - | - | - | 1.07 | 1.00 – 1.14 | 0.050 | |
| BMI × gender | - | - | - | - | - | - | - | - | - | 1.05 | 0.98 – 1.12 | 0.165 | |
| Age | -0.08 | -0.09 – -0.06 | <0.001 | -0.08 | -0.10 – -0.07 | <0.001 | 0.60 | 0.58 – 0.62 | <0.001 | 0.60 | 0.57 – 0.62 | <0.001 | |
| *Country (ref: Belgium)* | | | | | | | | | | | | | |
| Austria | 0.13 | 0.06 – 0.20 | <0.001 | 0.14 | 0.07 – 0.21 | <0.001 | 1.76 | 1.48 – 2.09 | <0.001 | 1.78 | 1.50 – 2.11 | <0.001 | |
| Czech Republic | 0.29 | 0.23 – 0.35 | <0.001 | 0.30 | 0.24 – 0.37 | <0.001 | 1.31 | 1.13 – 1.52 | <0.001 | 1.32 | 1.14 – 1.53 | <0.001 | |
| Denmark | -0.05 | -0.11 – 0.02 | 0.158 | -0.04 | -0.10 – 0.02 | 0.215 | 2.06 | 1.75 – 2.44 | <0.001 | 2.07 | 1.76 – 2.45 | <0.001 | |
| Estonia | 0.23 | 0.16 – 0.29 | <0.001 | 0.25 | 0.18 – 0.31 | <0.001 | 1.36 | 1.17 – 1.58 | <0.001 | 1.38 | 1.18 – 1.60 | <0.001 | |
| France | -0.09 | -0.15 – -0.03 | 0.006 | -0.09 | -0.15 – -0.02 | 0.009 | 1.08 | 0.94 – 1.26 | 0.282 | 1.09 | 0.94 – 1.26 | 0.261 | |
| Germany | 0.14 | 0.08 – 0.20 | <0.001 | 0.14 | 0.08 – 0.20 | <0.001 | 1.43 | 1.24 – 1.65 | <0.001 | 1.43 | 1.24 – 1.65 | <0.001 | |
| Israël | 0.12 | 0.01 – 0.23 | 0.037 | 0.13 | 0.01 – 0.24 | 0.027 | 0.92 | 0.72 – 1.19 | 0.524 | 0.93 | 0.72 – 1.19 | 0.558 | |
| Luxembourg | -0.19 | -0.26 – -0.13 | <0.001 | -0.21 | -0.27 – -0.14 | <0.001 | 0.73 | 0.63 – 0.84 | <0.001 | 0.72 | 0.62 – 0.83 | <0.001 | |
| Slovenia | 0.09 | -0.01 – 0.20 | 0.071 | 0.09 | -0.01 – 0.19 | 0.086 | 1.25 | 0.97 – 1.60 | 0.082 | 1.25 | 0.97 – 1.60 | 0.084 | |
| Italy | 0.14 | 0.06 – 0.22 | <0.001 | 0.15 | 0.07 – 0.22 | <0.001 | 1.67 | 1.39 – 2.01 | <0.001 | 1.68 | 1.40 – 2.02 | <0.001 | |
| Spain | 0.01 | -0.05 – 0.08 | 0.674 | 0.01 | -0.06 – 0.07 | 0.864 | 1.43 | 1.23 – 1.67 | <0.001 | 1.43 | 1.22 – 1.66 | <0.001 | |
| Sweden | -0.05 | -0.11 – 0.02 | 0.156 | -0.04 | -0.10 – 0.02 | 0.219 | 2.17 | 1.86 – 2.54 | <0.001 | 2.18 | 1.86 – 2.55 | <0.001 | |
| Switzerland | -0.18 | -0.25 – -0.11 | <0.001 | -0.18 | -0.25 – -0.11 | <0.001 | 2.17 | 1.83 – 2.58 | <0.001 | 2.17 | 1.83 – 2.58 | <0.001 | |
| IADL | 0.03 | 0.01 – 0.05 | 0.001 | 0.04 | 0.02 – 0.06 | <0.001 | 0.63 | 0.60 – 0.66 | <0.001 | 0.63 | 0.60 – 0.66 | <0.001 | |
| *Ability to make ends (ref: Easily)* | | | | | | | | | | | | | |
| Fairly easily | 0.12 | 0.08 – 0.15 | <0.001 | 0.12 | 0.09 – 0.15 | <0.001 | 0.85 | 0.78 – 0.92 | <0.001 | 0.85 | 0.79 – 0.92 | <0.001 | |
| With some  difficulty | 0.16 | 0.12 – 0.20 | <0.001 | 0.17 | 0.12 – 0.21 | <0.001 | 0.74 | 0.67 – 0.81 | <0.001 | 0.74 | 0.67 – 0.82 | <0.001 | |
| With great difficulty | 0.24 | 0.18 – 0.30 | <0.001 | 0.24 | 0.18 – 0.30 | <0.001 | 0.64 | 0.55 – 0.74 | <0.001 | 0.64 | 0.55 – 0.74 | <0.001 | |
| *Education (ref: Primary)* | | | | | | | | | | | | | |
| Secondary | -0.12 | -0.16 – -0.08 | <0.001 | -0.12 | -0.16 – -0.08 | <0.001 | 1.11 | 1.01 – 1.22 | 0.024 | 1.11 | 1.01 – 1.22 | 0.025 | |
| Tertiary | -0.25 | -0.30 – -0.20 | <0.001 | -0.25 | -0.30 – -0.20 | <0.001 | 1.19 | 1.06 – 1.34 | 0.004 | 1.19 | 1.05 – 1.34 | 0.005 | |
| *Occupation status (ref: High skill)* | | | | | | | | | | | | | |
| Low skill | 0.08 | 0.05 – 0.11 | <0.001 | 0.09 | 0.06 – 0.12 | <0.001 | 0.98 | 0.91 – 1.07 | 0.682 | 0.99 | 0.92 – 1.07 | 0.834 | |
| Never worked | 0.01 | -0.06 – 0.08 | 0.784 | 0.06 | -0.01 – 0.14 | 0.075 | 0.73 | 0.62 – 0.85 | <0.001 | 0.75 | 0.64 – 0.89 | 0.001 | |

*Note:* Model 3b included an interaction term between gender and social deprivation on BMI, Model 4b included interaction terms between gender and material deprivation and gender and BMI on physical activity Unstandardized b coefficients and odds-ratios (OR) and their 95% confidence intervals (95% CI) are reported. IADL: functional dependence in instrumental activities of daily living at baseline (Wave 5), was expressed as a continuous score ranging from 0 to 7 and was assessed in reference of seven IADL (using a map, preparing a hot meal, shopping for groceries, making telephone calls, taking medication, gardening or doing housework, and managing money).

**Table S6**. Estimates for all predictors in the models including material deprivation and wave of measurement as a confounding variable.

|  | **Model 1a (BMI as outcome)** | | | **Model 3a (BMI as outcome)** | | | **Model 2a (PA as outcome)** | | | **Model 4a (PA as outcome)** | | | |
| --- | --- | --- | --- | --- | --- | --- | --- | --- | --- | --- | --- | --- | --- |
| **Predictors** | **b** | **95% CI** | ***p*** | **b** | **95% CI** | ***p*** | **OR** | **95% CI** | ***p*** | **OR** | **95% CI** | | ***p*** |
| Intercept | 0.02 | -0.05 – 0.09 | 0.526 | 0.01 | -0.06 – 0.08 | 0.746 | 1.96 | 1.67 – 2.31 | **<0.001** | 1.95 | 1.66 – 2.30 | **<0.001** | |
| Wave (ref: Wave 8) | -0.02 | -0.06 – 0.02 | 0.331 | -0.02 | -0.06 – 0.02 | 0.399 | 0.99 | 0.90 – 1.09 | 0.833 | 0.99 | 0.91 – 1.09 | 0.886 | |
| Material deprivation | 0.06 | 0.04 – 0.08 | <0.001 | 0.04 | 0.02 – 0.06 | <0.001 | 0.88 | 0.84 – 0.92 | <0.001 | 0.87 | 0.83 – 0.91 | <0.001 | |
| BMI | - | - | - |  |  |  | 0.81 | 0.78 – 0.83 | <0.001 | 0.80 | 0.77 – 0.83 | <0.001 | |
| Gender (ref : men) | - | - | - | -0.14 | -0.17 – -0.12 | <0.001 | - | - | - | 0.88 | 0.82 – 0.94 | <0.001 | |
| Material deprivation × gender | - | - | - | 0.14 | 0.11 – 0.17 | <0.001 | - | - | - | 1.07 | 1.00 – 1.14 | 0.049 | |
| BMI × gender | - | - | - | - | - | - | - | - | - | 1.04 | 0.97 – 1.11 | 0.231 | |
| Age | -0.07 | -0.08 – -0.05 | <0.001 | -0.07 | -0.09 – -0.06 | <0.001 | 0.55 | 0.53 – 0.58 | <0.001 | 0.55 | 0.53 – 0.57 | <0.001 | |
| *Country (ref: Belgium)* | | | | | | | | | | | | | |
| Austria | 0.12 | 0.04 – 0.19 | 0.001 | 0.13 | 0.06 – 0.20 | <0.001 | 1.84 | 1.55 – 2.18 | <0.001 | 1.86 | 1.57 – 2.21 | <0.001 | |
| Czech Republic | 0.30 | 0.24 – 0.37 | <0.001 | 0.31 | 0.25 – 0.38 | <0.001 | 1.26 | 1.09 – 1.46 | 0.002 | 1.28 | 1.10 – 1.48 | 0.001 | |
| Denmark | -0.06 | -0.12 – 0.00 | 0.064 | -0.05 | -0.12 – 0.01 | 0.093 | 2.19 | 1.86 – 2.58 | <0.001 | 2.20 | 1.86 – 2.59 | <0.001 | |
| Estonia | 0.20 | 0.13 – 0.27 | <0.001 | 0.21 | 0.14 – 0.28 | <0.001 | 1.44 | 1.23 – 1.68 | <0.001 | 1.46 | 1.25 – 1.70 | <0.001 | |
| France | -0.09 | -0.16 – -0.03 | 0.005 | -0.09 | -0.15 – -0.02 | 0.007 | 1.13 | 0.97 – 1.31 | 0.105 | 1.13 | 0.98 – 1.32 | 0.094 | |
| Germany | 0.13 | 0.07 – 0.19 | <0.001 | 0.13 | 0.07 – 0.19 | <0.001 | 1.48 | 1.28 – 1.71 | <0.001 | 1.48 | 1.29 – 1.71 | <0.001 | |
| Israël | 0.13 | 0.02 – 0.25 | 0.020 | 0.14 | 0.03 – 0.26 | 0.012 | 0.82 | 0.64 – 1.06 | 0.124 | 0.83 | 0.65 – 1.06 | 0.136 | |
| Luxembourg | -0.20 | -0.26 – -0.13 | <0.001 | -0.20 | -0.26 – -0.14 | <0.001 | 0.76 | 0.66 – 0.88 | <0.001 | 0.76 | 0.66 – 0.88 | <0.001 | |
| Slovenia | 0.10 | -0.00 – 0.20 | 0.056 | 0.10 | -0.01 – 0.20 | 0.070 | 1.22 | 0.96 – 1.57 | 0.112 | 1.22 | 0.95 – 1.56 | 0.119 | |
| Italy | 0.12 | 0.04 – 0.20 | 0.002 | 0.13 | 0.05 – 0.21 | 0.001 | 1.90 | 1.57 – 2.29 | <0.001 | 1.91 | 1.58 – 2.30 | <0.001 | |
| Spain | 0.00 | -0.06 – 0.07 | 0.894 | 0.00 | -0.06 – 0.07 | 0.951 | 1.49 | 1.28 – 1.74 | <0.001 | 1.49 | 1.28 – 1.73 | <0.001 | |
| Sweden | -0.05 | -0.12 – 0.01 | 0.089 | -0.05 | -0.11 – 0.01 | 0.128 | 2.34 | 2.00 – 2.74 | <0.001 | 2.35 | 2.01 – 2.75 | <0.001 | |
| Switzerland | -0.19 | -0.26 – -0.12 | <0.001 | -0.19 | -0.26 – -0.12 | <0.001 | 2.39 | 2.01 – 2.85 | <0.001 | 2.39 | 2.01 – 2.85 | <0.001 | |
| *Ability to make ends (ref: Easily)* | | | | | | | | | | | | | |
| Fairly easily | 0.11 | 0.08 – 0.14 | <0.001 | 0.11 | 0.08 – 0.15 | <0.001 | 0.85 | 0.78 – 0.92 | <0.001 | 0.85 | 0.79 – 0.92 | <0.001 | |
| With some  difficulty | 0.12 | 0.07 – 0.17 | <0.001 | 0.13 | 0.08 – 0.17 | <0.001 | 0.75 | 0.67 – 0.83 | <0.001 | 0.75 | 0.68 – 0.84 | <0.001 | |
| With great difficulty | 0.17 | 0.10 – 0.25 | <0.001 | 0.18 | 0.10 – 0.25 | <0.001 | 0.63 | 0.53 – 0.74 | <0.001 | 0.63 | 0.53 – 0.74 | <0.001 | |
| *Education (ref: Primary)* | | | | | | | | | | | | | |
| Secondary | -0.13 | -0.17 – -0.09 | <0.001 | -0.13 | -0.17 – -0.09 | <0.001 | 1.17 | 1.07 – 1.29 | 0.001 | 1.17 | 1.07 – 1.28 | 0.001 | |
| Tertiary | -0.26 | -0.31 – -0.21 | <0.001 | -0.26 | -0.31 – -0.21 | <0.001 | 1.29 | 1.14 – 1.45 | <0.001 | 1.28 | 1.13 – 1.44 | <0.001 | |
| *Occupation status (ref: High skill)* | | | | | | | | | | | | | |
| Low skill | 0.08 | 0.05 – 0.11 | <0.001 | 0.10 | 0.06 – 0.13 | <0.001 | 0.95 | 0.87 – 1.02 | 0.163 | 0.96 | 0.88 – 1.04 | 0.278 | |
| Never worked | 0.03 | -0.04 – 0.10 | 0.473 | 0.08 | 0.01 – 0.15 | 0.035 | 0.65 | 0.55 – 0.75 | <0.001 | 0.68 | 0.58 – 0.80 | <0.001 | |

*Note:* Model 3a included an interaction term between gender and social deprivation on BMI, Model 4a included interaction terms between gender and material deprivation and gender and BMI on physical activity Unstandardized b coefficients and odds-ratios (OR) and their 95% confidence intervals (95% CI) are reported. Wave of measurement corresponds to the timepoint at which physical activity was last measured (i.e., Wave 7 or Wave 8).

**Table S7**. Estimates for all predictors in the models including social deprivation and wave of measurement as a confounding variable.

|  | **Model 1b (BMI as outcome)** | | | **Model 3b (BMI as outcome)** | | | **Model 2b (PA as outcome)** | | | **Model 4b (PA as outcome)** | | | |
| --- | --- | --- | --- | --- | --- | --- | --- | --- | --- | --- | --- | --- | --- |
| **Predictors** | **b** | **95% CI** | ***p*** | **b** | **95% CI** | ***p*** | **OR** | **95% CI** | ***p*** | **OR** | **95% CI** | | ***p*** |
| Intercept | -0.00 | -0.07 – 0.07 | 0.922 | -0.01 | -0.08 – 0.06 | 0.736 | 2.04 | 1.73 – 2.40 | <0.001 | 2.03 | 1.73 – 2.39 | <0.001 | |
| Wave (ref: Wave 8) | -0.02 | -0.06 – 0.02 | 0.429 | -0.01 | -0.05 – 0.03 | 0.519 | 0.98 | 0.89 – 1.07 | 0.636 | 0.98 | 0.89 – 1.08 | 0.688 | |
| Social deprivation | 0.05 | 0.03 – 0.06 | <0.001 | 0.04 | 0.02 – 0.06 | <0.001 | 0.79 | 0.77 – 0.82 | <0.001 | 0.79 | 0.76 – 0.82 | <0.001 | |
| BMI | - | - | - | - | - | - | 0.81 | 0.79 – 0.84 | <0.001 | 0.80 | 0.78 – 0.83 | <0.001 | |
| Gender (ref : men) | - | - | - | -0.15 | -0.17 – -0.12 | <0.001 | - | - | - | 0.89 | 0.83 – 0.95 | <0.001 | |
| Social deprivation × gender | - | - | - | 0.08 | 0.06 – 0.11 | <0.001 | - | - | - | 1.06 | 0.99 – 1.13 | 0.108 | |
| BMI × gender | - | - | - | - | - | - | - | - | - | 1.04 | 0.97 – 1.11 | 0.240 | |
| Age | -0.07 | -0.09 – -0.06 | <0.001 | -0.08 | -0.10 – -0.06 | <0.001 | 0.57 | 0.55 – 0.60 | <0.001 | 0.57 | 0.55 – 0.59 | <0.001 | |
| *Country (ref: Belgium)* | | | | | | | | | | | | | |
| Austria | 0.13 | 0.06 – 0.20 | <0.001 | 0.14 | 0.07 – 0.21 | <0.001 | 1.73 | 1.46 – 2.05 | <0.001 | 1.75 | 1.47 – 2.07 | <0.001 | |
| Czech Republic | 0.29 | 0.22 – 0.35 | <0.001 | 0.30 | 0.24 – 0.37 | <0.001 | 1.39 | 1.20 – 1.61 | <0.001 | 1.40 | 1.21 – 1.63 | <0.001 | |
| Denmark | -0.04 | -0.11 – 0.02 | 0.197 | -0.04 | -0.10 – 0.03 | 0.259 | 2.02 | 1.71 – 2.39 | <0.001 | 2.03 | 1.72 – 2.40 | <0.001 | |
| Estonia | 0.23 | 0.17 – 0.30 | <0.001 | 0.25 | 0.18 – 0.32 | <0.001 | 1.37 | 1.18 – 1.60 | <0.001 | 1.39 | 1.19 – 1.62 | <0.001 | |
| France | -0.09 | -0.16 – -0.03 | 0.006 | -0.09 | -0.15 – -0.02 | 0.009 | 1.14 | 0.99 – 1.33 | 0.074 | 1.15 | 0.99 – 1.33 | 0.068 | |
| Germany | 0.14 | 0.08 – 0.20 | <0.001 | 0.14 | 0.08 – 0.20 | <0.001 | 1.44 | 1.25 – 1.66 | <0.001 | 1.44 | 1.25 – 1.66 | <0.001 | |
| Israël | 0.13 | 0.02 – 0.24 | 0.022 | 0.14 | 0.03 – 0.25 | 0.016 | 0.87 | 0.68 – 1.12 | 0.281 | 0.88 | 0.68 – 1.12 | 0.298 | |
| Luxembourg | -0.20 | -0.27 – -0.13 | <0.001 | -0.21 | -0.28 – -0.15 | <0.001 | 0.80 | 0.69 – 0.93 | 0.003 | 0.79 | 0.69 – 0.92 | 0.002 | |
| Slovenia | 0.10 | -0.00 – 0.20 | 0.056 | 0.10 | -0.01 – 0.20 | 0.070 | 1.23 | 0.97 – 1.59 | 0.097 | 1.23 | 0.96 – 1.58 | 0.102 | |
| Italy | 0.14 | 0.06 – 0.22 | <0.001 | 0.15 | 0.07 – 0.23 | <0.001 | 1.75 | 1.46 – 2.12 | <0.001 | 1.76 | 1.46 – 2.13 | <0.001 | |
| Spain | 0.01 | -0.05 – 0.08 | 0.658 | 0.01 | -0.06 – 0.07 | 0.854 | 1.47 | 1.26 – 1.71 | <0.001 | 1.46 | 1.25 – 1.69 | <0.001 | |
| Sweden | -0.04 | -0.11 – 0.02 | 0.183 | -0.04 | -0.10 – 0.03 | 0.241 | 2.24 | 1.92 – 2.63 | <0.001 | 2.25 | 1.93 – 2.64 | <0.001 | |
| Switzerland | -0.18 | -0.25 – -0.11 | <0.001 | -0.18 | -0.25 – -0.11 | <0.001 | 2.27 | 1.91 – 2.70 | <0.001 | 2.27 | 1.91 – 2.70 | <0.001 | |
| *Ability to make ends (ref: Easily)* | | | | | | | | | | | | | |
| Fairly easily | 0.12 | 0.08 – 0.15 | <0.001 | 0.12 | 0.09 – 0.15 | <0.001 | 0.84 | 0.78 – 0.91 | <0.001 | 0.85 | 0.78 – 0.92 | <0.001 | |
| With some  difficulty | 0.16 | 0.12 – 0.20 | <0.001 | 0.17 | 0.13 – 0.21 | <0.001 | 0.71 | 0.65 – 0.79 | <0.001 | 0.72 | 0.65 – 0.79 | <0.001 | |
| With great difficulty | 0.25 | 0.19 – 0.31 | <0.001 | 0.25 | 0.19 – 0.31 | <0.001 | 0.58 | 0.50 – 0.67 | <0.001 | 0.58 | 0.50 – 0.67 | <0.001 | |
| *Education (ref: Primary)* | | | | | | | | | | | | | |
| Secondary | -0.12 | -0.16 – -0.08 | <0.001 | -0.13 | -0.17 – -0.09 | <0.001 | 1.14 | 1.04 – 1.25 | 0.004 | 1.14 | 1.04 – 1.25 | 0.005 | |
| Tertiary | -0.25 | -0.30 – -0.20 | <0.001 | -0.25 | -0.30 – -0.20 | <0.001 | 1.23 | 1.09 – 1.39 | 0.001 | 1.23 | 1.09 – 1.38 | 0.001 | |
| *Occupation status (ref: High skill)* | | | | | | | | | | | | | |
| Low skill | 0.08 | 0.05 – 0.11 | <0.001 | 0.09 | 0.06 – 0.12 | <0.001 | 0.97 | 0.89 – 1.05 | 0.399 | 0.98 | 0.90 – 1.06 | 0.539 | |
| Never worked | 0.02 | -0.05 – 0.09 | 0.641 | 0.07 | -0.00 – 0.14 | 0.051 | 0.68 | 0.58 – 0.80 | <0.001 | 0.72 | 0.61 – 0.84 | <0.001 | |

*Note:* Model 3b included an interaction term between gender and social deprivation on BMI, Model 4b included interaction terms between gender and material deprivation and gender and BMI on physical activity. Unstandardized b coefficients and odds-ratios (OR) and their 95% confidence intervals (95% CI) are reported. Wave of measurement corresponds to the timepoint at which physical activity was last measured (i.e., Wave 7 or Wave 8).
